# Supplementary material for: Mouse Ovarian Cancer Models Recapitulate the Human Tumor Microenvironment and Patient Response to Treatment
Source: Cell Rep. 2020 Jan 14;30(2):525–540.e7. doi: 10.1016/j.celrep.2019.12.034 (PMC6963791; doi:10.1016/j.celrep.2019.12.034)
Supplement: Document S1. Figures S1–S6 [file mmc1.pdf]

Cell Reports, Volume *30*

Supplemental Information

Mouse Ovarian Cancer Models Recapitulate the Human Tumor Microenvironment and Patient Response to Treatment

Eleni Maniati, Chiara Berlato, Ganga Gopinathan, Owen Heath, Panoraia Kotantaki, Anissa Lakhani, Jacqueline McDermott, Colin Pegrum, Robin M. Delaine-Smith, Oliver M.T. Pearce, Priyanka Hirani, Joash D. Joy, Ludmila Szabova, Ruth Perets, Owen J. Sansom, Ronny Drapkin, Peter Bailey, and Frances R. Balkwill

Figure S1

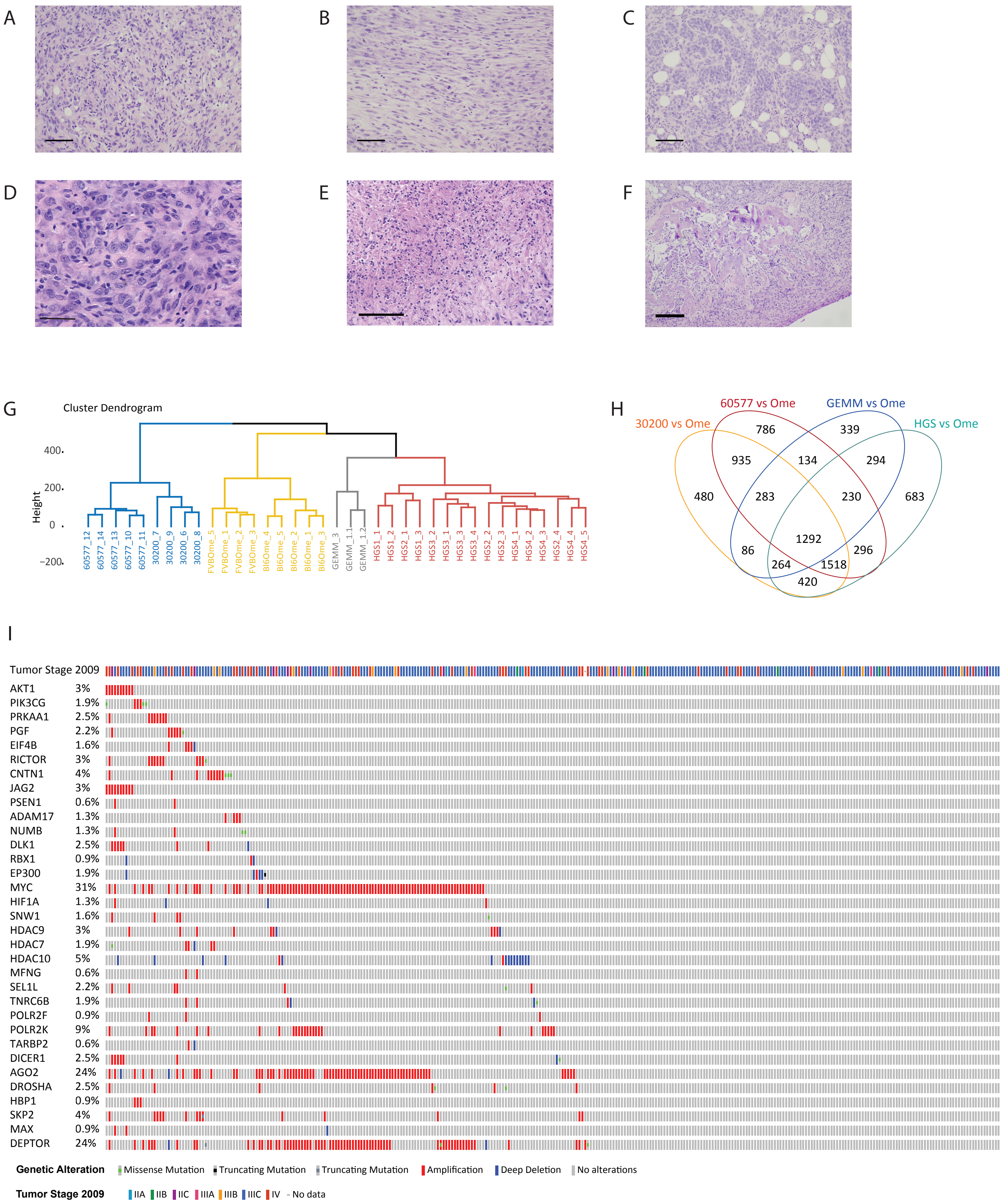

A
B
C
D
E
F
G
Cluster Dendrogram
Height
400
200
0
-200
60577_12
60577_14
60577_13
60577_10
60577_11
30200_7
30200_9
30200_6
30200_8
FVBOme_5
FVBOme_1
FVBOme_2
FVBOme_3
Bl6Ome_4
Bl6Ome_5
Bl6Ome_2
Bl6Ome_1
Bl6Ome_3
GEMM_3
GEMM_1.1
GEMM_1.2
HGS1_1
HGS1_2
HGS2_1
HGS1_3
HGS3_2
HGS3_1
HGS3_3
HGS3_4
HGS2_2
HGS2_3
HGS4_1
HGS4_2
HGS4_3
HGS2_4
HGS4_4
HGS4_5
H
60577 vs Ome
GEMM vs Ome
30200 vs Ome
HGS vs Ome
786
339
935
134
294
480
283
230
683
1292
86
296
264
1518
420
I
Tumor Stage 2009
AKT1 3%
PIK3CG 1.9%
PRKAA1 2.5%
PGF 2.2%
EIF4B 1.6%
RICTOR 3%
CNTN1 4%
JAG2 3%
PSEN1 0.6%
ADAM17 1.3%
NUMB 1.3%
DLK1 2.5%
RBX1 0.9%
EP300 1.9%
MYC 31%
HIF1A 1.3%
SNW1 1.6%
HDAC9 3%
HDAC7 1.9%
HDAC10 5%
MFNG 0.6%
SEL1L 2.2%
TNRC6B 1.9%
POLR2F 0.9%
POLR2K 9%
TARBP2 0.6%
DICER1 2.5%
AGO2 24%
DROSHA 2.5%
HBP1 0.9%
SKP2 4%
MAX 0.9%
DEPTOR 24%
Genetic Alteration
Missense Mutation
Truncating Mutation
Truncating Mutation
Amplification
Deep Deletion
No alterations
Tumor Stage 2009
IIA
IIB
IIC
IIIA
IIIB
IIIC
IV
No data

Figure S1. Histological and molecular characterisation of murine HGSOC models, related to Figure 1. **A-F** Tissue sections were derived from mouse model tumors and stained with hematoxylin and eosin **A**: HGS2, representation of tumour cells organised in sheets, scale bar set to 200µm; **B**: HGS3, representation of spindle-like morphology, scale bar set to 200µm; **C**: 60577, representation of tumour cells organized in nests, scale bar set to 200µm; **D**: HGS2, representation of pleomorphic cells with nuclei with prominent nucleoli, scale bar set to 50µm; **E**: HGS2, necrotic area, scale bar set to 200µm; **F**: HGS2, calcification, scale bar set to 200µm. **G**, Hierarchical cluster analysis of murine sample transcriptomes. **H**, Overlap of differentially-expressed genes (FDR < 0.05) in murine tumours vs normal omental samples. **I,** OncoPrint showing list of genes with high mutation frequency in TCGA and present in CNA regions in mouse models. Annotation of tumour stage has been included.

Figure S2

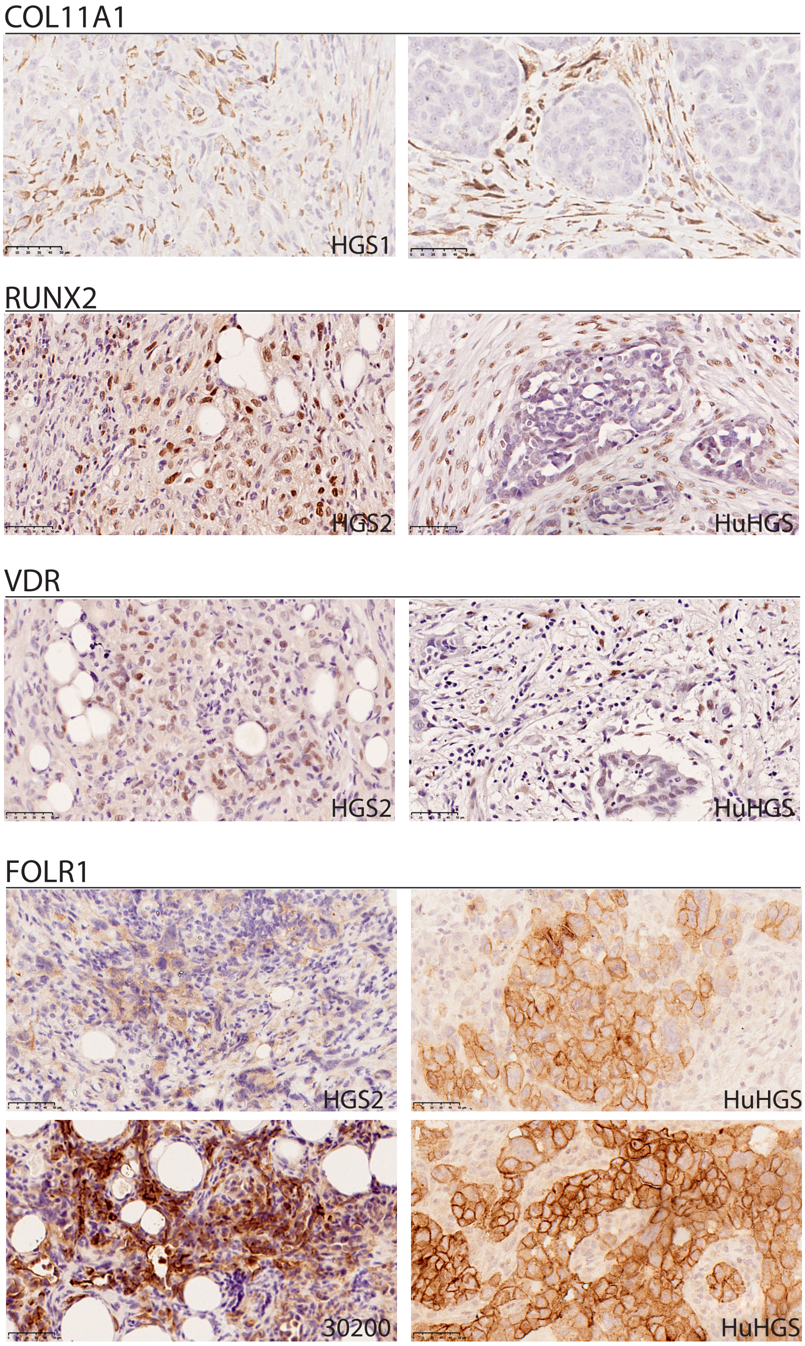
COL11A1
HGS1
RUNX2
HGS2
HuHGS
VDR
HGS2
HuHGS
FOLR1
HGS2
HuHGS
30200
HuHGS

Figure S2. Concordance of murine and human HGSOC tumor-associated proteins, related to Figure 2. **A,** IHC for COL11A1, RUNX2, VDR and FOLR1 staining. Representative images for HGS2 and HuHGS are depicted. For FOLR1, staining on the 30200 model, in which we observed the highest expression of the marker, has been included. Scale bars,100µm (COL11A1) and 50µm, (RUNX2, VDR, FOLR1).

Figure S3

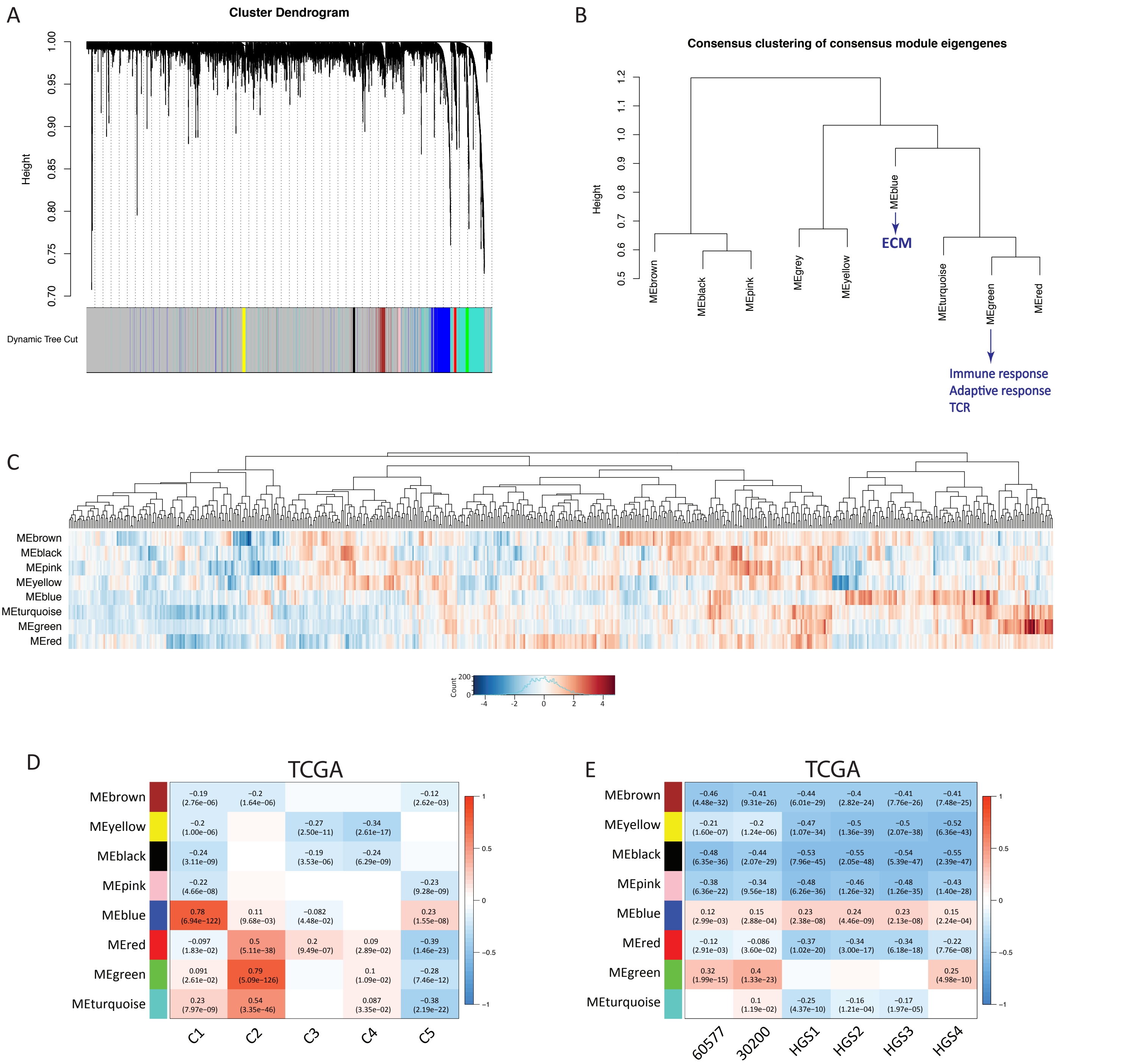
A
Cluster Dendrogram
Height
Dynamic Tree Cut
B
Consensus clustering of consensus module eigengenes
Height
MEbrown
MEblack
MEpink
MEgrey
MEyellow
MEblue
ECM
MEturquoise
MEgreen
MEred
Immune response
Adaptive response
TCR
C
MEbrown
MEblack
MEpink
MEyellow
MEblue
MEturquoise
MEgreen
MEred
Count
D
TCGA
MEbrown −0.19 (2.76e−06) −0.2 (1.64e−06) −0.12 (2.62e−03)
MEyellow −0.2 (1.00e−06) −0.27 (2.50e−11) −0.34 (2.61e−17)
MEblack −0.24 (3.11e−09) −0.19 (3.53e−06) −0.24 (6.29e−09)
MEpink −0.22 (4.66e−08) −0.23 (9.28e−09)
MEblue 0.78 (6.94e−122) 0.11 (9.68e−03) −0.082 (4.48e−02) 0.23 (1.55e−08)
MEred −0.097 (1.83e−02) 0.5 (5.11e−38) 0.2 (9.49e−07) 0.09 (2.89e−02) −0.39 (1.46e−23)
MEgreen 0.091 (2.61e−02) 0.79 (5.09e−126) 0.1 (1.09e−02) −0.28 (7.46e−12)
MEturquoise 0.23 (7.97e−09) 0.54 (3.35e−46) 0.087 (3.35e−02) −0.38 (2.19e−22)
C1 C2 C3 C4 C5
E
TCGA
MEbrown −0.46 (4.48e−32) −0.41 (9.31e−26) −0.44 (6.01e−29) −0.4 (2.82e−24) −0.41 (7.76e−26) −0.41 (7.48e−25)
MEyellow −0.21 (1.60e−07) −0.2 (1.24e−06) −0.47 (1.07e−34) −0.5 (1.36e−39) −0.5 (2.07e−38) −0.52 (6.36e−43)
MEblack −0.48 (6.35e−36) −0.44 (2.07e−29) −0.53 (7.96e−45) −0.55 (2.05e−48) −0.54 (5.39e−47) −0.55 (2.39e−47)
MEpink −0.38 (6.36e−22) −0.34 (9.56e−18) −0.48 (6.26e−36) −0.46 (1.26e−32) −0.48 (1.26e−35) −0.43 (1.40e−28)
MEblue 0.12 (2.99e−03) 0.15 (2.88e−04) 0.23 (2.38e−08) 0.24 (4.46e−09) 0.23 (2.13e−08) 0.15 (2.24e−04)
MEred −0.12 (2.91e−03) −0.086 (3.60e−02) −0.37 (1.02e−20) −0.34 (3.00e−17) −0.34 (6.18e−18) −0.22 (7.76e−08)
MEgreen 0.32 (1.99e−15) 0.4 (1.33e−23) 0.25 (4.98e−10)
MEturquoise 0.1 (1.19e−02) −0.25 (4.37e−10) −0.16 (1.21e−04) −0.17 (1.97e−05)
60577 30200 HGS1 HGS2 HGS3 HGS4

Figure S3. ICGC and TCGA consensus WGCNA, related to Figure 3. **A,** WGCNA consensus analysis was performed using the ovarian ICGC and TCGA expression datasets and consensus clusters of co-regulated genes are presented as a dendrogram. Colors show different modules (gene programs). **B**, Cluster dendrogram of consensus module eigenvalues (ME) illustrates clustering of programs associated with ECM (MEblue) and immune response (MEgreen) pathways. **C**, Heatmap of consensus ME across TCGA samples (N = 593). **D**, Heatmap of association of C1–C5 classification. Positive associations are shown in red and negative are shown in blue. Pearson's r and p-values are indicated in the fields where a significant association was observed ($p < 0.05$). **E**, Heatmap of association of differentially-expressed gene scores in mouse models. Positive associations are shown in red and negative are shown in blue. Pearson's r and p-values are indicated in the fields where a significant association was observed ($p < 0.05$).

Figure S4

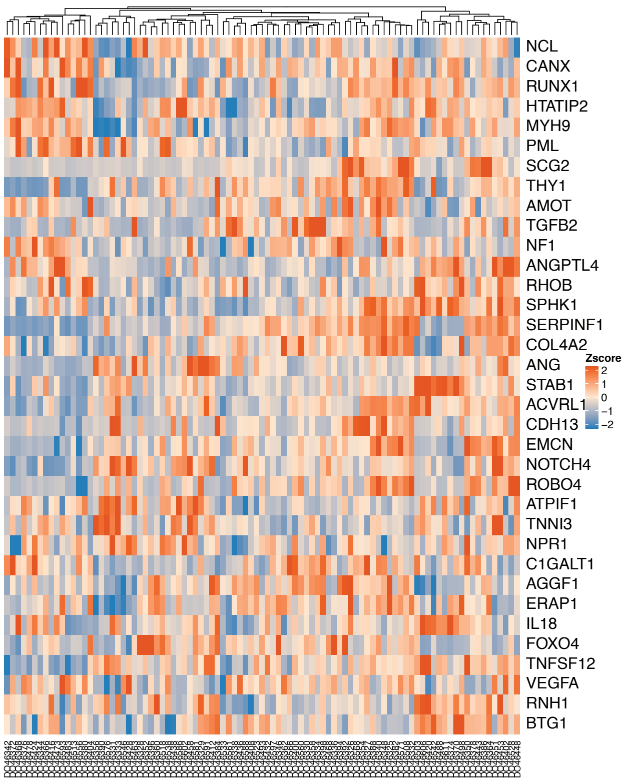

NCL
CANX
RUNX1
HTATIP2
MYH9
PML
SCG2
THY1
AMOT
TGFB2
NF1
ANGPTL4
RHOB
SPHK1
SERPINF1
COL4A2
ANG
STAB1
ACVRL1
CDH13
EMCN
NOTCH4
ROBO4
ATPIF1
TNNI3
NPR1
C1GALT1
AGGF1
ERAP1
IL18
FOXO4
TNFSF12
VEGFA
RNH1
BTG1
Zscore
2
1
0
−1
−2

Figure S4, related to Figure 4. Heatmap illustrating expression of angiogenesis-associated genes (N = 93) across primary HGSOC tumors in the ICGC dataset.

Figure S5

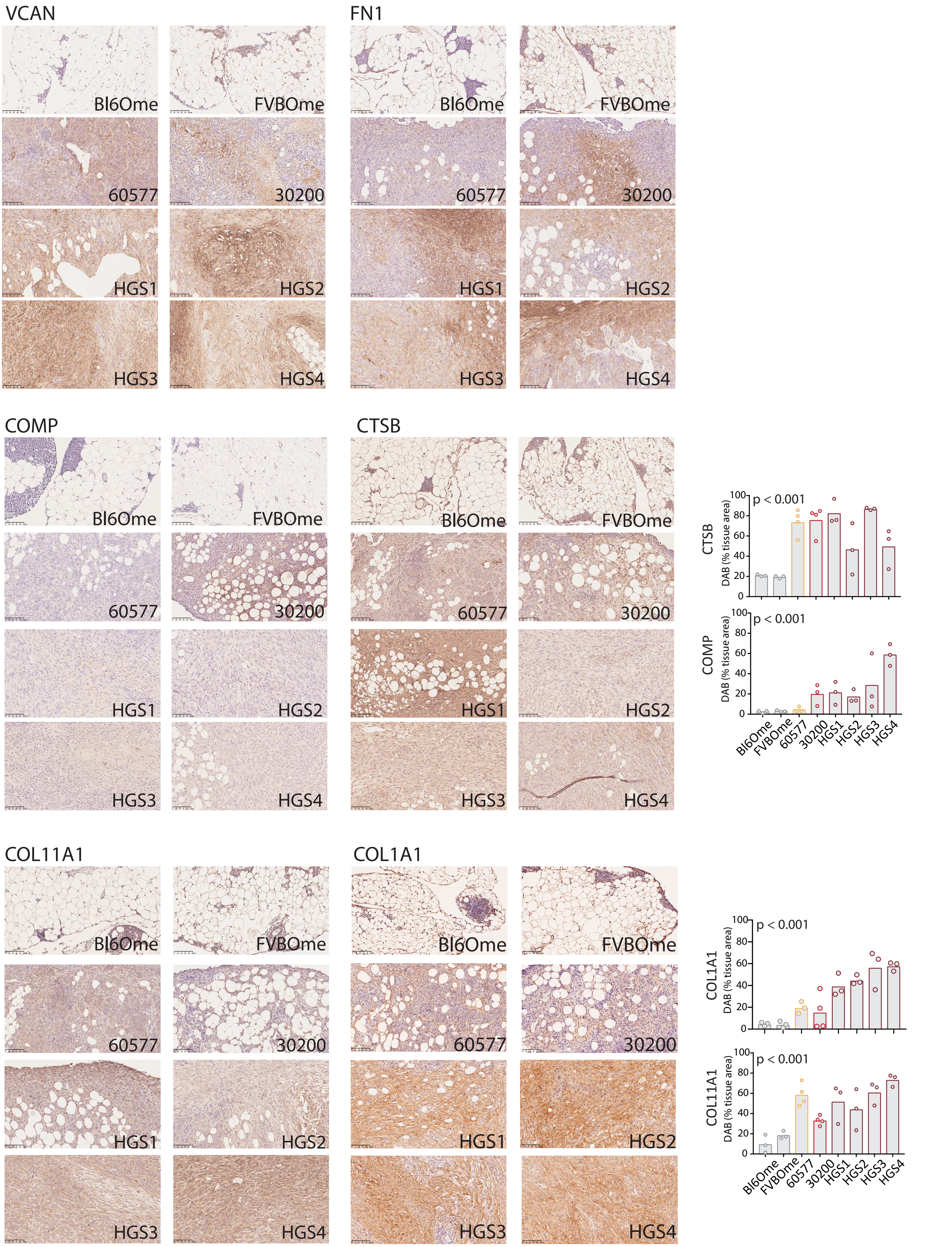

VCAN
FN1
COMP
CTSB
COL11A1
COL1A1
Bl6Ome
FVBOme
60577
30200
HGS1
HGS2
HGS3
HGS4
p < 0.001
CTSB
COMP
COL1A1
COL11A1
DAB (% tissue area)
0
20
40
60
80
100

Figure S5. Matrix index proteins in HGSOC murine models, related to Figure 5. IHC for VCAN, FN1, COMP, CTSB, COL11A1, COL1A1. Representative images all six HGSOC murine models and normal omental tissues is shown. Scale bars,100µm. Barplots illustrate the result of digital analysis and quantification of % positive area by Definiens Tissue Studio®. P-values correspond to one-way ANOVA. Each dot represents a tumor from an individual mouse.

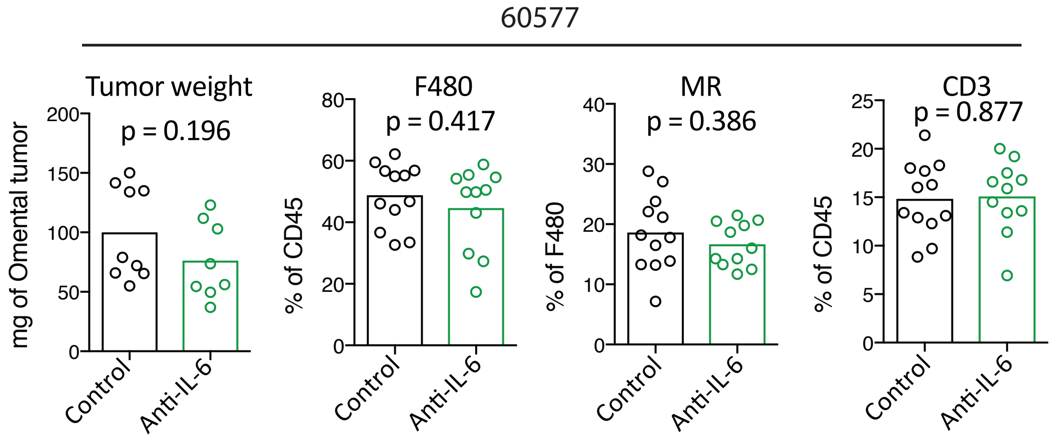
60577
Tumor weight
p = 0.196
mg of Omental tumor
0
50
100
150
200
Control
Anti-IL-6
F480
p = 0.417
% of CD45
0
20
40
60
80
Control
Anti-IL-6
MR
p = 0.386
% of F480
0
10
20
30
40
Control
Anti-IL-6
CD3
p = 0.877
% of CD45
0
5
10
15
20
25
Control
Anti-IL-6

Figure S6. Anti-IL6 treatment of the 60577 murine model, related to Figure 6. Mice injected with 60577 cells were treated with isotype control or anti-IL-6 2 mg/kg i.p. twice weekly, starting 10 days after cell injection until endpoint. The immune infiltrates were analyzed by flow cytometry. Student's t-test values are depicted on the barplots. Each dot represents a tumor from an individual mouse.
