# Supplementary material for: Mouse Ovarian Cancer Models Recapitulate the Human Tumor Microenvironment and Patient Response to Treatment
Source: Cell Rep. 2020 Jan 14;30(2):525–540.e7. doi: 10.1016/j.celrep.2019.12.034 (PMC6963791; doi:10.1016/j.celrep.2019.12.034)
Supplement: Document S2. Article plus Supplemental Information [file mmc9.pdf]

Cell Reports

Article

Mouse Ovarian Cancer Models Recapitulate the Human Tumor Microenvironment and Patient Response to Treatment

Graphical Abstract

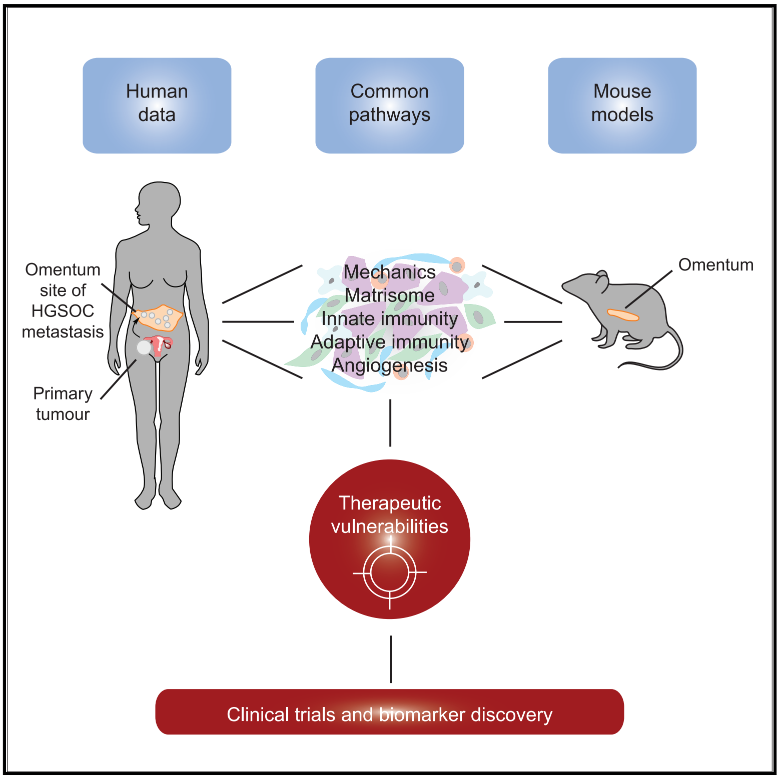

Authors

Eleni Maniati, Chiara Berlato, Ganga Gopinathan, ..., Ronny Drapkin, Peter Bailey, Frances R. Balkwill

Correspondence

f.balkwill@qmul.ac.uk

In Brief

Maniati et al. show how orthotopic transplantable mouse ovarian cancers with appropriate genotypes develop microenvironments that replicate many features of human primary ovarian tumors and metastases. Molecular features of the mouse tumors combined with machine learning may allow the identification of patients who are most likely to respond to specific therapies.

Highlights

- Mouse ovarian cancers replicate the human ovarian tumor microenvironment
- Stromal, immune, and malignant cell targets in common
- Evidence for distinct therapeutic vulnerabilities in individual models
- Models may help identify patient subgroups likely to respond to specific therapies

Maniati et al., 2020, Cell Reports *30*, 525–540
January 14, 2020 © 2019 The Author(s).
https://doi.org/10.1016/j.celrep.2019.12.034

CellPress

Cell Reports
Article

OPEN ACCESS
CellPress

Mouse Ovarian Cancer Models Recapitulate the Human Tumor Microenvironment and Patient Response to Treatment

Eleni Maniati,[1,9] Chiara Berlato,[1,9] Ganga Gopinathan,[1,9] Owen Heath,[1] Panoraia Kotantaki,[1] Anissa Lakhani,[1] Jacqueline McDermott,[2] Colin Pegrum,[1] Robin M. Delaine-Smith,[1] Oliver M.T. Pearce,[1] Priyanka Hirani,[1] Joash D. Joy,[1] Ludmila Szabova,[3] Ruth Perets,[4] Owen J. Sansom,[5,6] Ronny Drapkin,[7] Peter Bailey,[5,8,9] and Frances R. Balkwill[1,9,10,*]
[1]Barts Cancer Institute, Queen Mary University of London, London EC1M 6BQ, UK
[2]University College Hospital, UCLH Cellular Pathology, 11-20 Capper Street, London WC1E 6JA, UK
[3]Center for Advanced Preclinical Research, Frederick National Laboratory for Cancer Research at the National Cancer Institute-Frederick, Frederick, MD, USA
[4]Rambam Health Care Campus, Technion - Israel Institute of Technology, Haifa, Israel
[5]Cancer Research UK Beatson Institute, Garscube Estate, Switchback Road, Glasgow, G61 1BD, UK
[6]Institute of Cancer Sciences, University of Glasgow, Garscube Estate, Switchback Road, Glasgow, G61 1QH, UK
[7]Penn Ovarian Cancer Research Center, University of Pennsylvania Perelman School of Medicine, Philadelphia, PA 19104, USA
[8]Department for Surgical Research, Universitätsklinikum Erlangen, Erlangen, Germany
[9]These authors contributed equally
[10]Lead Contact
*Correspondence: f.balkwill@qmul.ac.uk
https://doi.org/10.1016/j.celrep.2019.12.034

SUMMARY

Although there are many prospective targets in the tumor microenvironment (TME) of high-grade serous ovarian cancer (HGSOC), pre-clinical testing is challenging, especially as there is limited information on the murine TME. Here, we characterize the TME of six orthotopic, transplantable syngeneic murine HGSOC lines established from genetic models and compare these to patient biopsies. We identify significant correlations between the transcriptome, host cell infiltrates, matrisome, vasculature, and tissue modulus of mouse and human TMEs, with several stromal and malignant targets in common. However, each model shows distinct differences and potential vulnerabilities that enabled us to test predictions about response to chemotherapy and an anti-IL-6 antibody. Using machine learning, the transcriptional profiles of the mouse tumors that differed in chemotherapy response are able to classify chemotherapy-sensitive and -refractory patient tumors. These models provide useful pre-clinical tools and may help identify subgroups of HGSOC patients who are most likely to respond to specific therapies.

INTRODUCTION

Human tumors comprise a complex mixture of malignant cells, immune, and other stromal cells regulated by a dynamic network of soluble mediators and adhesion molecules. All of these components interact in an abnormal extracellular matrix (ECM), often referred to as the tumor matrisome (Socovich and Naba, 2018). Not only is this tumor microenvironment (TME) critical for the growth and spread of human cancers but also the non-malignant components are important targets for immunological and other biological therapies (Binnewies et al., 2018; Foster et al., 2018; Mantovani et al., 2017).

We recently conducted multi-layered TME profiling of evolving omental metastases of human high-grade serous ovarian cancer (HGSOC), describing gene and protein profiles that are associated with tissue stiffness, extent of disease, and cellularity (Pearce et al., 2018). During this analysis, we defined a 22-gene matrisome signature, the Matrix Index, which predicted the extent of disease, tissue remodeling, and tissue stiffness. When we interrogated publicly available transcriptional datasets from >9,000 primary tumor biopsies, we found that a high Matrix Index distinguished patients with shorter overall survival, not only in ovarian cancer but also in 12 other human cancer types. This suggested that there may be a common host matrix response in human primary and metastatic cancers (Pearce et al., 2018). As regulators of these matrisome molecules may be important therapeutic targets across many different cancer types, pre-clinical models that replicate the malignant matrisome and immune landscapes are of critical importance.

However, it is not clear whether TMEs of murine cancer models sufficiently replicate their human counterparts. Differences between the human and mouse TMEs may compromise pre-clinical studies of novel immune and other biological therapies and their successful translation to clinical trials. Many murine cancer models may not have appropriate oncogenic mutations and may be grown in immunocompromised animals or in unsuitable anatomical sites; others grow too rapidly for a TME to develop fully or too slowly for pre-clinical studies to be feasible.

HGSOC is typified by ubiquitous *TP53* mutations/deletions (Ahmed et al., 2010; The Cancer Genome Atlas Research Network, 2011). Homologous DNA repair defects, especially

 © 2019 The Author(s).
This is an open access article under the CC BY license (http://creativecommons.org/licenses/by/4.0/).

BRCA1 or *-2* alterations are found. The phosphatidylinositol 3-kinase (PI3K) pathways, through phophatase and tensin homolog (PTEN) deletion and other mechanisms, and retinoblastoma (RB) pathways are also often altered (The Cancer Genome Atlas Research Network, 2011). Most other mutations are of low frequency, but copy number alterations (CNAs) are frequent and complex (Macintyre et al., 2018). Previous mouse models of HGSOC include peritoneal xenografts from human cell lines of uncertain origin or syngeneic transplantable models such as ID8 that do not possess appropriate mutations. Genetic engineering of the ID8 model by CRISPR/Cas9 has resulted in a more suitable transplantable model with Trp53 and Brca2 deletions (Walton et al., 2016). There are several useful genetically engineered mouse models (GEMMs) of HGSOC (Perets et al., 2013; Zhai et al., 2017), but their mixed backgrounds and complex breeding programs make them unsuitable for studies of tumor immunity and immunotherapy (Stuckelberger and Drapkin, 2018).

To find models of the HGSOC TME that replicate the immune and matrisome components of human disease, we studied a range of murine models that have disease-relevant genetic mutations, are transplantable, and are relatively slow growing. We chose models that develop metastases in one of the most common sites found in women, the omentum, a metastatic site that we have extensively characterized in patient biopsies (Böhm et al., 2016; Montfort et al., 2017; Pearce et al., 2018). Four of these models were generated in our laboratory from tumors that developed in a GEMM (Perets et al., 2013) that we backcrossed onto a B6 background, and two were cell lines that had been originally established from tumors from GEMM tumors generated by adenoviral transduction (Szabova et al., 2014). We then conducted multi-level molecular and cellular profiling of murine peritoneal metastases in these six different transplantable mouse models to determine their suitability as models for human HGSOC.

Here, we demonstrate that many of the biomechanical, cellular, and molecular features of human HGSOC are replicated in the murine tumors with significant correlations in mRNA expression profiles, innate and adaptive immune responses, tissue modulus, and matrisome components. Further highlighting the utility of these models as avatars of human disease, we find that the mouse models exhibit significant differences and distinct vulnerabilities in their TMEs, reflecting the heterogeneity of human HGSOC biopsies. Using this model platform, we conducted proof-of-concept studies that demonstrate the potential of this repertoire of models for pre-clinical studies and found that the transcriptional profile of chemotherapy-responsive murine tumors translates to patients, suggesting that these mouse models could help identify sub-groups of patients who would most benefit from a specific treatment.

RESULTS

Mouse HGSOC Models

As >98% of HGSOC are *TP53* null or have *TP53* mutations and ~50% have defects in homologous double-stranded DNA (dsDNA) repair, we focused on models with key genotypes. Details of the genetic mutations, latency, and distribution of metastases of the individual models are summarized in Figure 1A and Table S1A. Cell lines 30200 and 60577, originally developed from GEMMs of serous ovarian cancer (Szabova et al., 2014), which had been engineered to be *Trp53*$^{-/-}$, *Brca1*$^{-/-}$, and expressed TAg_{121} (the N-terminal domain of SV40 T antigen), under control of the cytokeratin 18 promoter, to inactivate the tumor suppressor function of *Rb*. These models are syngeneic in FVB mice and originate from the ovarian surface epithelium. Following intraperitoneal (i.p.) injection, they produced extensive omental and peritoneal metastases, with established disease detected at ~20 weeks (30200) and 6 weeks (60577).

We also used another GEMM, developed by Perets et al. (2013), with *Pax8*-Cre driving inducible inactivation of *Brca2*, *Trp53*, and *Pten* (*Brca2*$^{-/-}$*;Trp53*$^{-/-}$*;Pten*$^{-/-}$ or *Brca2*$^{+/-}$*; Trp53*$^{-/-}$*;Pten*$^{-/-}$) in the fallopian tube. We extensively backcrossed these various GEMMs to the B6 background and established four polyclonal tumor cell lines, HGS1–4, from fallopian tube, ovary, or peritoneal tumors that developed in the induced backcrossed mice (details in Table S1A). When injected i.p., all of the HGS cell lines produced omental tumors and metastases to splenoportal fat, lesser omentum, and mesentery, with extensive disease established by 12–20 weeks (Figure 1B). Other metastatic sites were ovaries, fat surrounding the reproductive tract, liver, and diaphragm.

Tumors from the six cell lines had serous histology (Figure 1B) presenting as high-grade carcinomas arranged in solid sheets, with cells displaying spindle-like morphology (Figures S1A and S1B). Nests and papillae were occasionally present (Figure S1C). The malignant cells were pleomorphic with a high nuclear:cytoplasmic ratio and frequent mitoses (Figure S1D). The nuclei were irregular, and nucleoli were variably prominent (Figure S1D). Apoptosis was conspicuous, and some tumors exhibited necrosis (Figure S1E). There was occasional dystrophic calcification and evidence of muscle invasion (Figures 1B and S1F). All of these features are consistent with high-grade serous carcinoma.

Transcriptomic Analysis of Murine Tumors

Using RNA sequencing (RNA-seq), we analyzed the transcriptome of peritoneal tumors from the six models plus three "primary" tumors from the backcrossed GEMM (Perets et al., 2013) and normal omentum from FVB and B6 mice. We chose the omentum because it is a distinct structure that is easy to identify and dissect in the mouse. Moreover, omentum is a major site of metastasis in human disease (Nieman et al., 2011), and we had already conducted extensive multi-level analyses of human HGSOC omenta (Pearce et al., 2018). Accordingly, the majority of mouse tumors studied were from the omentum.

We analyzed RNA-seq data from three to five tumors per model, harvested when mice with extensive peritoneal disease reached a humane endpoint. A total of 14,201 protein-coding genes were sufficiently detected. Principal-component analysis (PCA) segregated the samples into three groups (Figure 1C). Control FVB and B6 omenta were in a distinct cluster from the FVB tumors 60577 and 30200. Tumors from the GEMM and HGS1–4 were grouped into a third cluster. A cluster dendrogram confirmed the distinct groups and close association of the HGS tumors with the GEMM (Figure S1G). We observed sub-clustering of HGS1, 3, and 4, while HGS2 was interspersed among

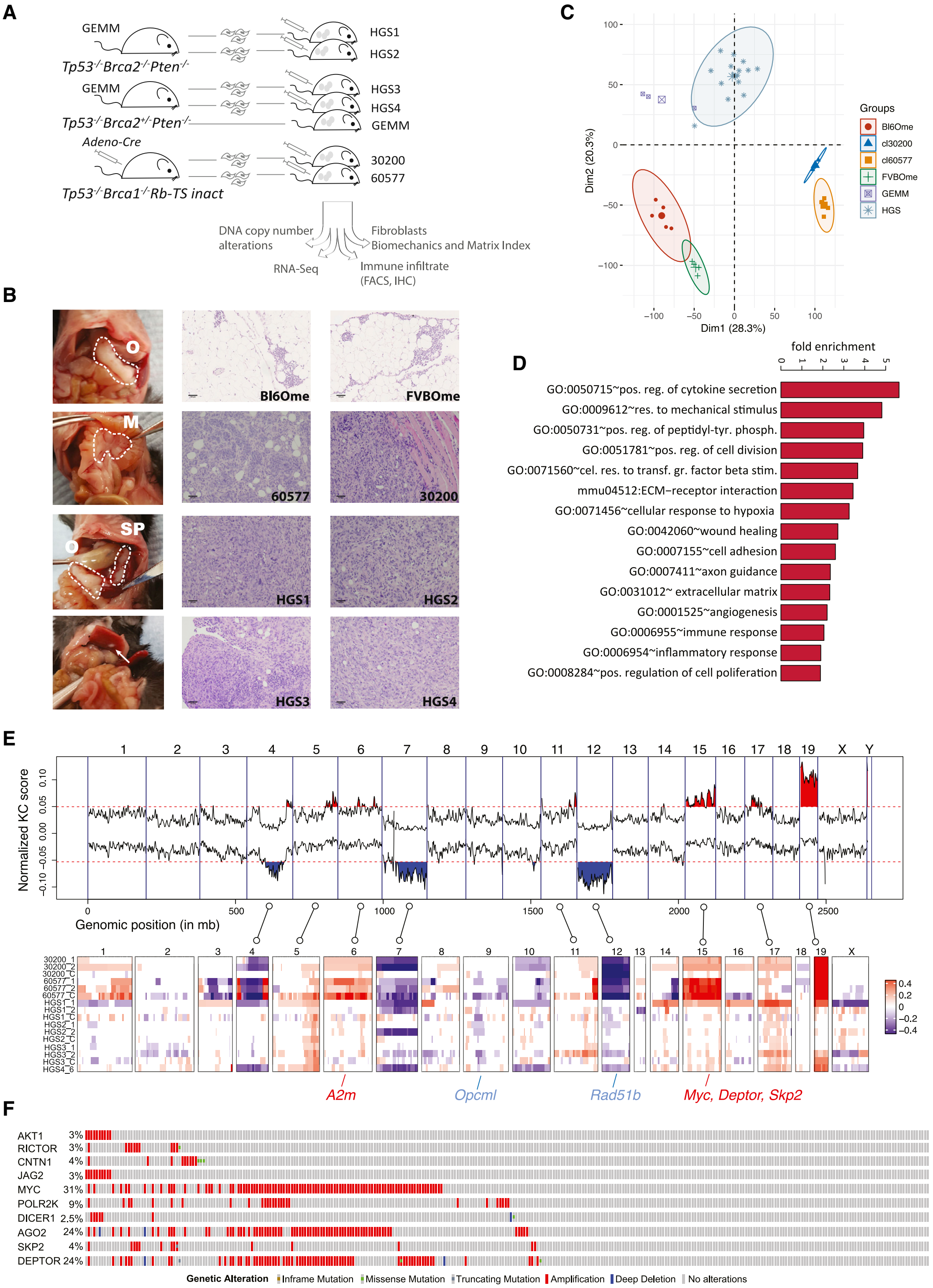

Figure 1. Characterization of Murine HGSOC Models

(A) Overview of murine models and the analyses conducted.

(B) Left panel: gross anatomy of the tumor distribution in the peritoneal cavity of a mouse injected with the HGS2 cell line and culled at a humane endpoint. Omental (O), mesenteric (M), and splenoportal (SP) tumor deposits are highlighted with a dashed line. A metastasis to the liver surface is indicated by an arrow.

(legend continued on next page)

HGS1 and 4, indicating that despite similarity among the HGS models, they retain some distinct transcriptomic features.

A total of 1,292 transcribed genes were significantly expressed (false discovery rate [FDR] < 0.05) in tumors from each of the models compared with normal omentum (Figure S1H). Enrichment analysis of these revealed common pathways altered in all of the murine peritoneal tumors compared with normal omentum, including TME-related pathways such as regulation of cytokine secretion, response to mechanical stimulus, transforming growth factor β (TGF-β) stimulation, hypoxia, ECM-receptor interactions, wound healing, immune and inflammatory responses, and angiogenesis (Figure 1D). As expected, other enriched pathways—for instance, cell proliferation, chromosome segregation, and development—were also featured (see complete list in Table S1B).

This analysis demonstrates that the TMEs of the individual mouse models exhibit common and distinct molecular features and that the enrichment of key pathways associated with immune response and the matrisome are over-represented in the transcriptomes.

Comparison of CNA in Mouse and Human Tumors

The Cancer Genome Atlas (TCGA) and International Cancer Genome Consortium (ICGC) sequencing have identified recurrent copy number alteration (CNAs) in HGSOC (Macintyre et al., 2018). To establish whether concordant CNAs existed between human disease and the mouse models, we extracted DNA from mouse tumors and cell lines and performed array comparative genomic hybridization (aCGH) copy number analysis. This identified genomic regions of recurrent gain and loss and included some of the top 20 significant recurrent CNAs in the TCGA database of human HGSOC (Figure 1E; Table S1C). Regions showing consistent amplification included *Myc* and genes associated with RNA processing, the PI3K pathway, the mammalian target of rapamycin (mTOR), and NOTCH signaling (Figures 1F and S1I) in agreement with findings in the original GEMM (Perets et al., 2013).

Comparison of Transcriptomes of Human and Mouse HGSOC Metastases

Having established concordance between human HGSOC tumors and the mouse models at the genomic level, we compared the transcriptomic profiles of established murine tumors with pre-treatment omental metastases from nine HGSOC patients. There was a significant correlation between the expression levels of orthologous genes in the murine tumors and human tumors (Figure 2A). Figure 2A also shows strong similarities between the 30200 and 60577 tumor lines and among the four HGS models and their respective GEMM tumors, as expected from Figure 1. The top concordant gene expression patterns between human and mouse peritoneal tumors are shown in Figure 2B (full list in Table S2A). Of particular interest was *COL11A1*, one of the six upregulated molecules in the Matrix Index (Pearce et al., 2018) that was also identified in a human pan-cancer gene signature of activated fibroblasts (Jia et al., 2016). We also noted enhanced expression of *RUNX2*, a transcription factor shared by a majority of genes in the Matrix Index (Pearce et al., 2018). Other commonly expressed genes of interest were the *VDR*, identified as a master transcription factor of stellate cells in pancreatic ductal adenocarcinoma (PDAC) (Sherman et al., 2014), and *FOLR1*, which is commonly overexpressed in ovarian cancer and a target for immunotherapy (Bergamini et al., 2016). Protein expression of these genes was confirmed by immunohistochemistry (IHC) (Figure S2). Using IHC, COL11A1, RUNX2, and VDR were primarily detected in the stroma, with some malignant cell positivity. FOLR1 was highly expressed on malignant cells.

We then looked in more detail at signaling pathways and found strong correlations between murine and human tumors in terms of the p53 and DNA-damage repair pathways; the mTOR, ErbB, and Hippo pathways; and cell-cell communication and tumor necrosis factor receptor 2 (TNFR2) non-canonical nuclear factor κB (NF-κB) signaling (Figure 2C). Several other common pathways included FoxO, nucleotide metabolism, estrogen signaling, semaphorin interactions, ECM, cell-junction organization, c-type lectin receptors, adherens junctions, and deactivation of β-catenins (Table S2B).

Biological processes significantly represented in both the mouse and human peritoneal tumors included wound healing, ECM organization and disassembly, immune response, angiogenesis, and malignant cell signaling pathways (Figure 2D).

In conclusion, metastases from human and murine tumors shared many common pathways and processes related to both the malignant cells and their interactions within the TME. Having identified common pathways between human and mouse omental metastases, we wanted to see how mouse omental metastases compared with primary HGSOC tumors.

Analysis of Mouse and Human Tumor Transcriptomes

To further characterize transcriptional programs preserved between mouse and human HGSOC tumor transcriptomes, we performed weighted correlation transcriptional network analysis (WGCNA) (Langfelder and Horvath, 2008; Bailey et al., 2016) on the ICGC HGSOC dataset of primary pre-treatment tumors (Patch et al., 2015). This analysis identified 18 coordinately expressed gene programs associated with distinct biological pathways and/or processes (Figure 3A; Tables S3A–S3G). The gene programs were significantly enriched for immune cell-specific genes associated with B cell and $CD8^+$ signatures or with macrophage genes and T cell co-inhibition (Figure 3B). We also identified a program enriched for antigen processing and

Center and right panels: tissue sections were derived from normal omenta (Bl6Ome and FVBOme) and mouse model tumors (60577, 30200, HGS1, HGS2, HGS3, and GEMM) and stained with hematoxylin and eosin (scale bars, 100 μm).
(C) Unsupervised clustering of RNA-seq sample groups by principal-component analysis.
(D) Significantly enriched Gene Ontology (GO) terms and pathways ($p < 0.001$) in the common 1,292 differentially expressed genes.
(E) Genomic alterations found in murine HGSOC. Copy number losses and gains are shown in blue and red, respectively. Key orthologous genes, frequently altered in human HGSOC, are indicated with blue for losses and red for gains.
(F) OncoPrint showing genes with high mutation frequency in TCGA and present in CNA regions in mouse models.

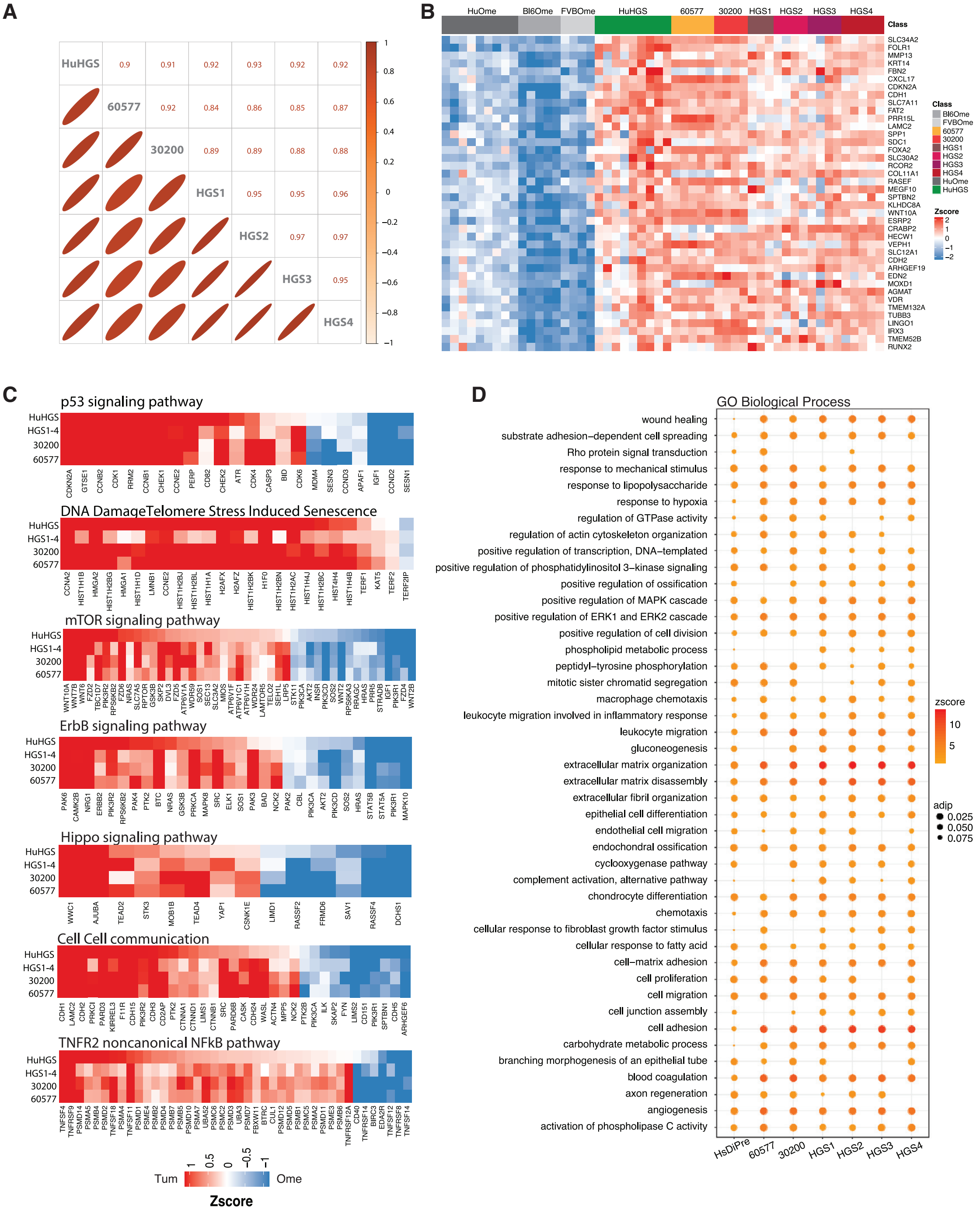

Figure 2. Comparison of Murine HGSOC and Human Omental Metastasis Transcriptomes

(A) Upper triangle: Pearson correlation coefficients based on the average expression (reads per kilobase million [RPKM]) of 12,127 orthologous genes in pairwise alignments of human omental tumors and mouse model tumors. Lower triangle: the diameter of the ellipses is proportional to the correlation coefficient; thinner ellipses correspond to higher correlation coefficients.

(B) Top concordantly upregulated genes in murine HGSOC and human omental tumors (FDR < 0.05).

(C and D) Key signaling pathways (C) identified by pathway analysis through Gaussian graphical models (clipper, pathway threshold p < 0.05), concordant in human and all mouse model tumors and biological processes (D) and significantly altered in both murine and human HGSOC (hypergeometric test p < 0.05). Cycle diameter proportional to adjusted p value; color corresponds to pathway *Z* score.

(A–D) 30200, HGS2, and HGS3, n = 4; 60577 and HGS4, n = 5; HGS1, n = 3; FVBOme, n = 4; Bl6Ome, n = 5. For human samples, n = 9 normal/adjacent omenta and n = 9 omental tumors.

OPEN
ACCESS
CellPress

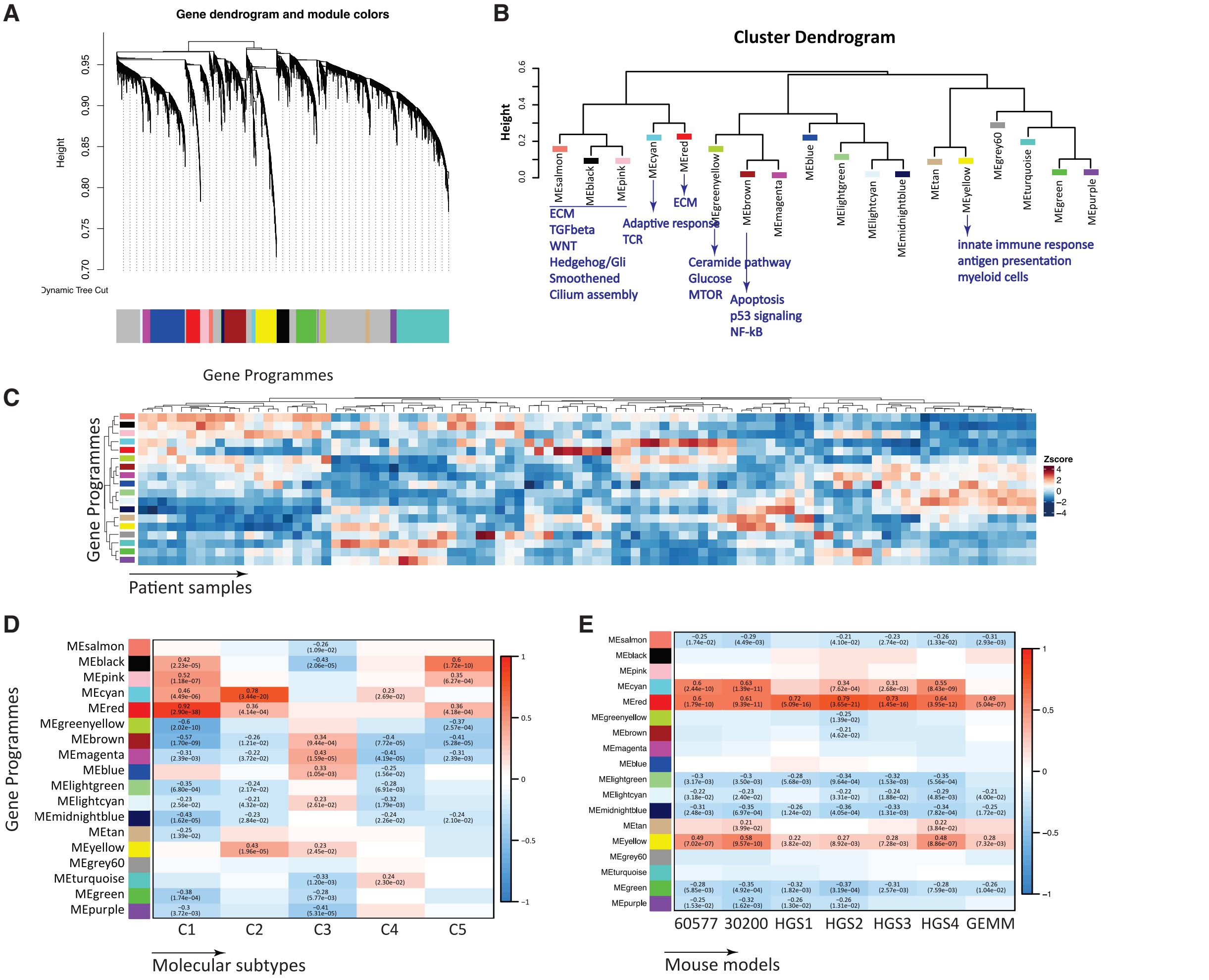

Figure 3. Comparison of Murine HGSOC and ICGC Ovarian Transcriptomes

(A) Weighted correlation network analysis (WCNA) of human ICGC transcriptional HGSOC dataset showing clusters of co-regulated genes as a dendrogram. Colors show different modules (gene programs).

(B) Cluster dendrogram of module eigenvalues (MEs) illustrates clustering of programs associated with ECM, immune response, or tumor-related signaling pathways.

(C) Heatmap of MEs across ICGC samples (n = 93).

(D) Heatmap of association of C1–C5 classification. Positive associations are shown in red and negative associations are shown in blue. Pearson's r and p values are indicated in the fields where a significant association was observed ($p < 0.05$).

(E) Heatmap of association of differentially expressed gene scores in mouse models. Positive associations are shown in red and negative associations are shown in blue. Pearson's r and p values are indicated in the fields where a significant association was observed ($p < 0.05$).

presentation, including high expression of Toll-like receptor genes (e.g., TLR4/7/8/PDL2, CSF1R) (Figure 3B). As expected, there was heterogeneity in the expression patterns of transcriptional programs across patient tumors (Figure 3C).

Next, we correlated the 18 gene programs with the C1–C5 molecular signatures identified in primary human HGSOC biopsies (Leong et al., 2015). C1 represents the mesenchymal subtype, and it strongly correlated with the ECM-enriched program (MEred, Figures 3B and 3D). C2 represents the immunoreactive subtype associated with the immune gene programs (MEcyan and MEyellow, Figures 3B and 3D). C3–C5 showed associations with other distinct sets of programs.

We then asked whether there was any association between 18 identified gene programs in the human primary tumors and the mouse tumors. ECM and immune response processes were the most significantly preserved gene programs across all of the models (Figure 3E). The HGS tumors associated more strongly with the ECM program (MEred) compared with the 30200 and 60577 tumors. However, the 30200 and 60577 tumors associated more strongly with the immune programs than the HGS models. Thus, despite the similarities between the mouse models, we were able to identify a number of cell line-specific TME characteristics. These results were independently verified using a consensus network obtained from the

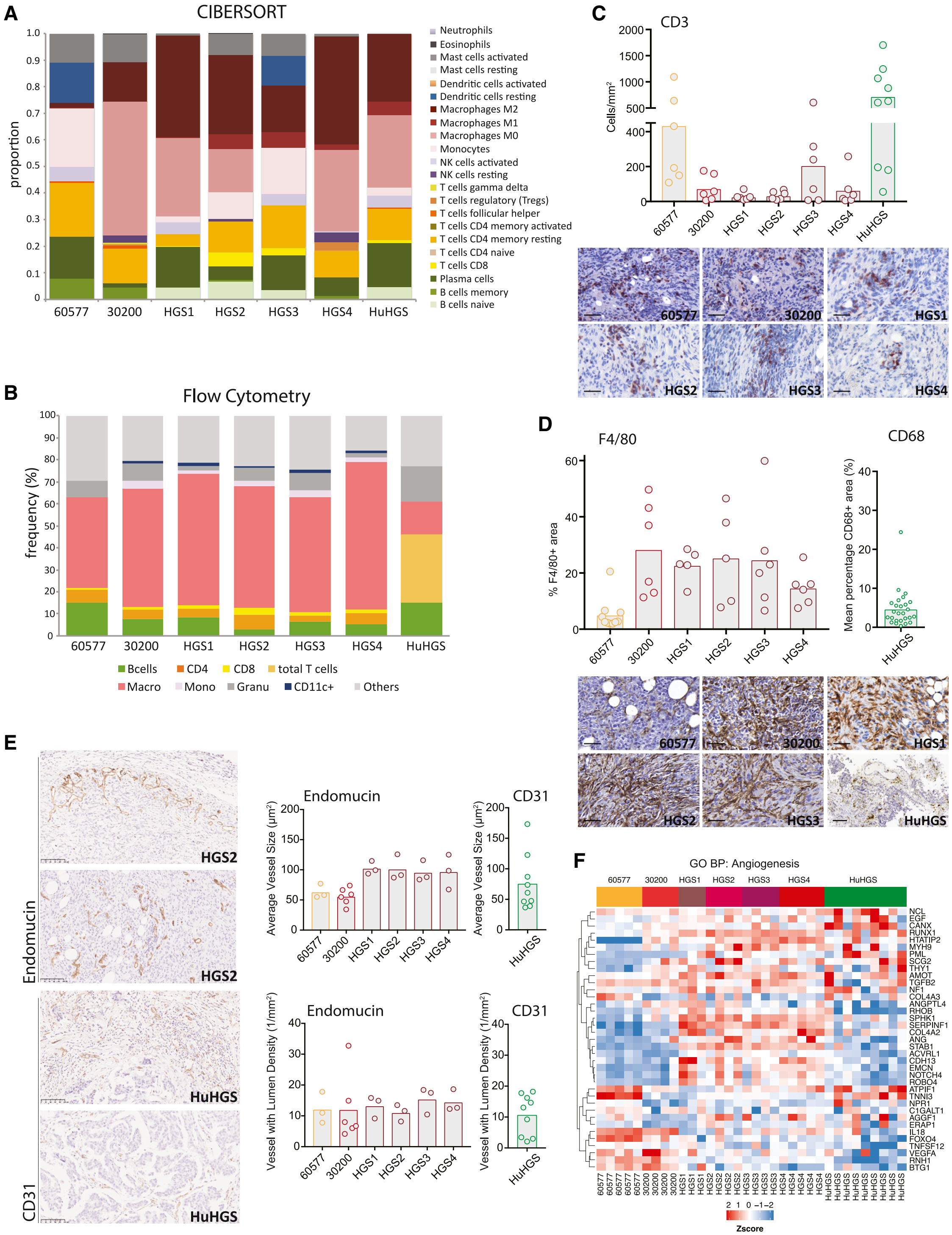

Figure 4. Immune Cells and Vasculature of the Murine and Human HGSOC

(A) Proportions of immune cell populations estimated from murine tumors and human omental metastases using CIBERSORT. 30200, HGS2, and HGS3, n = 4; 60577 and HGS4, n = 5; HGS1, n = 3; human omental tumors, n = 9. Median values are depicted.

(B) Flow cytometric analysis of the immune infiltrate of peritoneal tumors, close to endpoint, from mice injected with 60577, 30200, or HGS cell lines. B cells: $CD45^+$ $CD19^+$, CD4 cells: $CD45^+$ $CD3^+$ $CD4^+$, CD8 cells: $CD45^+$ $CD3^+$ $CD8^+$, monocytes: $CD45^+$ $CD11b^+$ $Ly6C^+$, macrophages: $CD45^+$, $CD11b^+$ $F4/80^+$ ($Ly6C/G^-$), granulocytes: $CD45^+$ $CD11b^+$ $Ly6G^+$, $CD11c^+$ cells: $CD45^+$ $CD11b^+$ $CD11c^+$ ($F4/80^-$ $Ly6C/G^-$). A similar analysis of the immune infiltrate in the diseased omentum from patients who underwent upfront surgery is shown for comparison (HuHGS).

(legend continued on next page)

TCGA ovarian transcriptomic data (Figure S3; Tables S4A–S4E).

Our data indicate that the mouse models share transcriptional patterns that are present in human HGSOC TMEs and primarily replicate the mesenchymal and immunoreactive human HGSOC subtypes.

Immune Cell Profiles of the Murine and Human HGSOC Tumors

To identify immune cell types and/or phenotypes associated with murine tumors or pre-treatment human omental metastases, we used CIBERSORT (Newman et al., 2015) to interrogate the transcriptomic data (Figure 4). According to the CIBERSORT analysis of the orthologous genes, all of the murine and human tumors contained significant populations of B cells, $CD4^+$ and $CD8^+$ T cells, monocytes, and macrophages (Figure 4A). Genes associated with resting dendritic cells, neutrophils, and natural killer (NK) cells were variably expressed. The profile of HGS1 most closely resembled human HGSOC metastases.

We then conducted flow cytometry on dissociated murine and human tumors to validate these results (Figure 4B). There were detectable populations of macrophages, monocytes, B cells, granulocytes, and $CD4^+$ T cells in the murine tumors with small populations of $CD8^+$ T cells and $CD11c^+$ dendritic cells. Heavily diseased human HGSOC omental samples had higher numbers of T cells and fewer myeloid cells than the murine tumors. We also assessed the density of T cells by IHC for $CD3^+$ T cells (Figure 4C) using Definiens Tissue Studio software. There was a variability in $CD3^+$ T cell density, which reflected the variability in the human omental metastases also seen in other studies of primary HGSOC (Zhang et al., 2018) and our published data on HGSOC omental metastases (Böhm et al., 2016; Pearce et al., 2018). The range was similar between mouse and human tumors, but overall, there were fewer $CD3^+$ T cells in the mouse tumors compared with the human samples ($p < 0.001$), although there was no difference between 60577 mouse tumors and the human tumors. Similarly, using IHC, we compared the density of F4/80$^+$ and $CD68^+$ macrophages in murine tumors and human omental metastases, respectively (Figure 4D). In agreement with the CIBERSORT RNA analysis, 60577 tumors had the lowest density of F4/80$^+$ cells, which was particularly interesting as they had the highest $CD3^+$ T cell density of all of the murine tumors. F4/80$^+$ cell densities were similar across the rest of the murine tumors but, with the exception of 60577 tumors, were at a higher density than the levels in the human omental metastases studied here ($p < 0.001$). There was no significant difference between 60577 and the human tumors.

Vasculature of Murine and Human HGSOC Tumors

As the vasculature of HGSOC is also an important therapeutic target (Oza et al., 2015), we quantified blood vessels in the murine omental tumors using Definiens Tissue Studio after IHC for endomucin and compared this to the density of $CD31^+$ blood vessels in human HGSOC omental metastases (Figure 4E). Both the average size and density of vessels with lumens were similar in murine and human omental metastases. IHC images (Figure 4E) show similar vessel structures and distribution in the reactive stroma around malignant areas (top images) and within the malignant areas (bottom images). Figure 4F shows the Gene Ontology biological pathway heatmap for angiogenesis in murine and human tumors. Here, the pattern of expression of angiogenic genes in the human biopsies seems to be a mix of the different murine tumors, and while 60577 seems to have a distinct gene expression pattern, this did not affect vessel density or size. The pattern of angiogenic gene expression was also variable in the primary HGSOC tumors (Figure S4).

Matrisome, Matrix Index, and Fibroblasts

As one of the aims of this study was to determine whether mouse tumors reproduce the prognostic matrisome gene expression patterns we found in human HGSOC (Pearce et al., 2018), we studied the pattern of matrisome gene expression in the mouse and human omental tumor biopsies and normal omenta. This analysis showed enhanced expression of collagens, matrisome glycoproteins, proteoglycans, ECM regulators, and ECM-secreted factors in both mouse and human tumors compared with control omenta (Figure 5A). There were also differences between the mouse models, especially between the HGS tumors and 60577 or 30200. In general, the murine tumors recapitulated our findings in human tumors, as shown by an increased expression of genes across all of the matrisome classes. Murine 60577 and 30200 tumors were unusual in upregulating more genes associated with small-molecule-secreted factors (shown in ECM-secreted factors and ECM regulators), rather than larger fibular components (e.g., collagens, proteoglycans), which were more similar to normal tissue (Figure 5A).

We then asked whether the murine tumors had similar values for the prognostic Matrix Index that we previously identified in the human omental metastases, primary HGSOC, and 12 other human solid cancer datasets (Pearce et al., 2018). The 22 Matrix Index genes (Table S5) were all expressed the mouse tumors. However, levels varied between low in 60577 to high in HGS1, thus showing variations in the Matrix Index (Figure 5B, left panel) that were similar to the range of values seen in diseased pre-treatment human omental HGSOC metastases (Figure 5B, right panel).

We then used mechanical indentation (Delaine-Smith et al., 2016) to measure tissue modulus (a measure of material stiffness independent of sample dimension). Tissue modulus values of the mouse tumors were one to two orders of magnitude higher than the normal human or mouse omentum (Figure 5C, left panel) and

(C) Quantification of the number of $CD3^+$ cells/mm^2 by IHC on peritoneal tumors from mice injected with 60577, 30200, or HGS cell lines. Quantification of $CD3^+$ cells in biopsies from patients (HuHGS) is shown for comparison. Representative images are shown; scale bars set to 100 μm.

(D) Quantification of the percentage of an area positive for F4/80 by IHC on peritoneal tumors from mice injected with 60577, 30200, or HGS cell lines. Quantification of $CD68^+$ area in patient biopsies is included for comparison. Representative images are shown; scale bars set to 100 μm.

(E) IHC for endomucin (mouse) and CD31 (human) staining, quantified using the Definiens Tissue Studio platform with the blood vessel detection feature. Each dot represents a tumor from an individual mouse or human. Representative images for HGS2 and HuHGS are depicted at left. Scale bars, 100 μm.

(F) Heatmap illustrating Gene Ontology biological process angiogenesis gene expression across mouse models and human omental tumors.

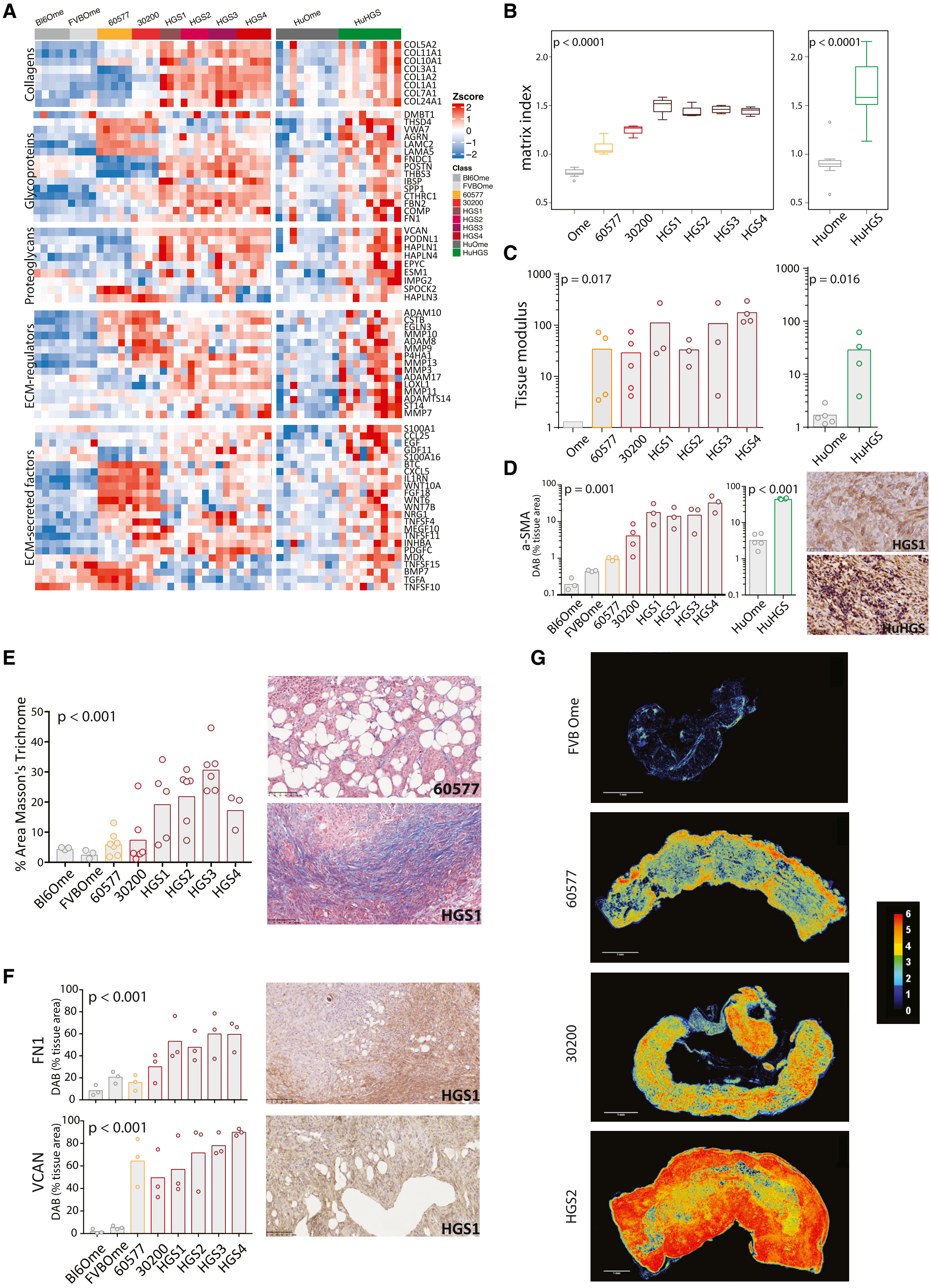

Figure 5. Matrisome, Matrix Index, and Stiffness of Murine and Human HGSOC

(A) Heatmap of orthologous matrisome genes, grouped by matrisome class, in the mouse and human peritoneal datasets compared to normal omenta (Student's t test, $p < 0.05$).

(legend continued on next page)

were in a range similar to that of diseased pre-treatment human HGSOC omentum (Figure 5C, right panel) values we have recently published (Pearce et al., 2018).

In our previous study of human HGSOC metastases, we found a high correlation between the density of fibroblastic cells positive for α-smooth muscle actin (αSMA) and the tissue modulus of the sample. In keeping with this finding, mouse tumors with the highest levels of αSMA were among the stiffer tumors with a higher Matrix Index (Figures 5B and 5D).

There were six matrisome molecules whose upregulation was associated with disease score and high tissue modulus in the human Matrix Index: fibronectin 1 (FN1), versican (VCAN), collagens 1A1 and 11A1 (COL1A1 and COL11A1), cathepsin B (CTSB), and cartilage oligometric matrix protein (COMP) (Pearce et al., 2018). All six were detected by IHC in mouse tumors but at varying levels. Figure 5E shows that Masson's trichrome staining for collagen followed a similar pattern to the data from Figures 5A–5D. Figure 5F shows the quantification of FN1 and VCAN staining in mouse tumors. The other four matrisome proteins were also detected by IHC (for representative images, see Figure S5). Overall, the strongest staining in all of the tumors was seen with FN1, VCAN, and CTSB, and the weakest for COMP.

Using sequential sections from the murine tumors, we then constructed a tissue matrisome heatmap of these six proteins using color deconvolution (Schneider et al., 2012; Ruifrok and Johnston, 2001). In some tumor areas, all six proteins were at high density. In Figure 5G, this is shown as absence (black) or the co-localization *in situ* of one (dark blue), two (cyan), three (green), four (yellow), five (orange), or six (red) matrisome molecules. HGS2 tumors had the highest number and areas of co-located molecules.

Overall, we find significant similarities between the mouse peritoneal tumors, human omental metastases, and primary human pre-treatment HGSOC tumors, especially in relation to immune and matrisome components. We believe that the models described above could be useful for the pre-clinical evaluation of TME-targeted therapies. However, each of our models had distinct characteristics and vulnerabilities, which are influenced by the mutations, tissue of origin, strain background, and genomic instability. We hypothesized that these "vulnerabilities" could be exploited therapeutically and that our HGSOC models would exhibit a range of different responses that reflect the range seen in patients.

Exploiting the Individual Vulnerabilities of the Six HGSOC Models

To investigate the above hypothesis and assess the suitability of our models for pre-clinical studies, we used the RNA-seq data to define in more detail the differences between the mouse models using the normal mouse omenta as controls. As expected, fatty acid biosynthesis and adipocytokine signaling expression pathways were significantly higher in the normal omenta compared to omental tumors. Conversely, Myc, cell-cycle, and Tp53 pathways were more dominant in the tumors compared to the normal omenta (Figure 6A). Of particular interest were significantly higher levels of cell-cycle pathways in the 60577 tumors compared to all of the other tumors, higher expression of the interleukin-6 (IL-6) pathway in the 30200 tumors, and higher expression of matrisome and integrin pathways in the HGS tumors (Figure 6A). Were the gene expression differences associated with response to therapy?

To test this hypothesis, we looked in more detail at the DNA replication gene expression heatmap, and based on the data shown in Figure 6B and the immune cell profiles in Figure 4C, we predicted that established 60577 tumors would be more responsive to carboplatin chemotherapy than HGS2. Survival of 60577-bearing mice was increased 5-fold by platinum treatment compared to <2-fold for HGS2-bearing mice (Figure 6C).

Next, we wanted to test a treatment that may modify the TME. In view of our previous pre-clinical and clinical data on IL-6 as a TME regulator in HGSOC (Coward et al., 2011; Stone et al., 2012), we looked in more detail at IL-6-regulated genes. We found that the 30200 tumors had the highest expression of genes in this pathway, while HGS2 and 60577 were lower (Figure 6D). We treated established tumors from three different models with anti-murine IL-6, and only in 30200 did we find significant modulation of the TME after treatment with an anti-IL-6 antibody. Specifically, after treatment for 5–7 weeks, there was a significant decrease in F4/80^{+} macrophages. Concomitant with this decrease in tumor-associated macrophages (TAMs), we noted a significant increase in CD3^{+} T cells. There was also a trend toward a decrease in macrophage mannose receptor expression in 30200 omental tumors (Figure 6E), suggesting a decline in tumor-promoting TAMs. Mice in the anti-IL-6-treated groups first reached the endpoint 5 weeks after the isotype-control mice, and there were long-term survivor mice in the treated group when the experiment was terminated at 56 weeks (Figure 6E).

(B) Matrix Index of murine and human peritoneal HGSOC and normal omenta. 30200, HGS2 and HGS3, n = 4; 60577 and HGS4, n = 5; HGS1 n = 3; FVBOme n = 4; Bl6Ome n = 5. For human samples, n = 9 normal/adjacent omenta and n = 9 omental tumors.

(C) Tissue modulus of murine and human peritoneal HGSOC and normal omenta. Each dot represents a tumor from an individual mouse or patient. p values correspond to the Kruskal-Wallis test for mouse data and the Mann-Whitney *U* test for human data.

(D) Fibroblast content of murine and human peritoneal HGSOC and normal omenta was assessed by IHC for αSMA staining and quantified using the Definiens Tissue Studio platform. p values correspond to one-way ANOVA. Each dot represents a tumor from an individual mouse. A representative image for HGS1 is depicted at right. Scale bar, 50 μm.

(E) Masson's trichrome staining was performed on all HGSOC model tumors and on normal omenta. Bar plot illustrates the result of digital analysis and the quantification of the percentage of positive area by the Definiens Tissue Studio. Representative images for 60577 and HGS1 are depicted at right. Scale bars, 100 μm. p values correspond to one-way ANOVA. Each dot represents a tumor from an individual mouse.

(F) IHC for FN1 and VCAN staining quantified using the Definiens Tissue Studio platform. p values correspond to one-way ANOVA. Each dot represents a tumor from an individual mouse. Representative images for HGS1 are depicted at right. Scale bars, 100 μm.

(G) Construction of tissue matrisome heatmaps for models of HGSOC. Serial IHC images were color deconvoluted, overlaid, and pseudo-colored using ImageJ to highlight areas that were rich (red) or poor (black) in ECM. Expression hotspots for all six ECM molecules are shown in red, whereas areas expressing one to five ECM molecules are presented with the different colors on the key map shown at right. Scale bars, 1 mm.

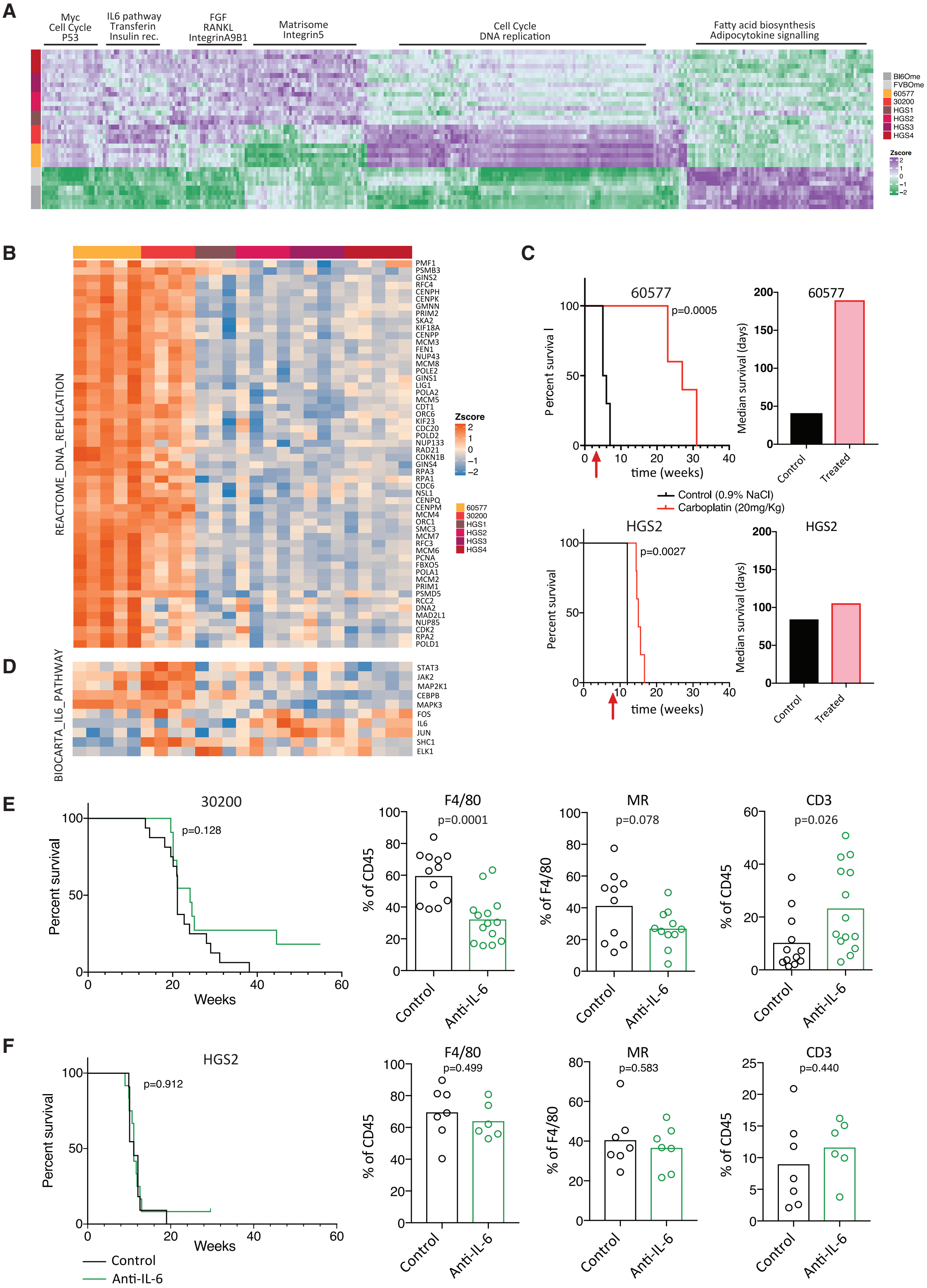

Figure 6. Therapeutic Vulnerabilities of HGSOC Models

(A) Single-sample gene set enrichment analysis (GSEA) was performed for the murine HGSOC transcriptomes. Heatmap illustrates pathway scores with distinct expression patterns in 30200, 60577, HGS tumors, and normal omenta (FDR < 0.05). 30200, HGS2, and HGS3, n = 4; 60577 and HGS4, n = 5; HGS1, n = 3; FVBOme, n = 4; Bl6Ome, n = 5.

(legend continued on next page)

There was no effect on macrophages or T cells in the omental tumors in similar experiments conducted on established HGS2 tumors (Figure 6F) and established 60577 tumors and no effect on survival in HGS2-bearing mice (survival not assessed in the 60577 model) (Figure S6).

Multivariate Classification of Patient Response Based on Mouse Model Data

Finally, we asked whether the mouse model data had relevance for the chemotherapy responses of HGSOC patients. Comparing the pre-treatment transcriptomes of the carboplatin-sensitive 60577 tumors with less sensitive HGS2 tumors, we identified genes that were differentially expressed (FDR < 0.0001, log fold change >|3|). Of these, 687 had human orthologs in the ICGC dataset of primary pre-treatment HGSOC tumors (Figure 7A; Table S6A). As chemotherapy outcomes are available for ICGC patients, we applied a multivariate classification tool (classyfire) (Chatzimichali and Bessant, 2016) that provides a state-of-the-art open source pipeline for the construction of robust classification models and implements support vector machine (SVM) ensemble classifiers with automated bootstrap training and permutation testing. We used classyfire to assess the relation between the expression pattern of the 687 genes and patient responses, as recorded in the ICGC dataset (Figure 7B).

classyfire correctly predicted responses in 70% of the ICGC patients. This value was an average across 50 SVM models within the ensemble, each of which was trained using 100 bootstrap repetitions (Figure 7C). Figure 7D illustrates that a stable classification ensemble, in which the accuracy of individual classifiers averaged out, was achieved after ~40 SVMs, which justifies choosing 50 SVMs in this analysis. Statistical significance, assessed by permutation testing with the number of permutations set to 100, indicated that the accuracy of the dataset was significantly higher than random chance ($p < 0.001$). Enrichment analysis showed that within the 687 gene patterns derived from the mouse tumors, patient resistance to chemotherapy was significantly associated with ECM organization, cell adhesion, and collagen catabolism and organization (Figure 7E; Table S6B). Our analysis indicates that transcriptional features of the mouse models that associate with chemotherapy response are also present in humans and are predominantly related to the tumor microenvironment.

DISCUSSION

As there are multiple immune and other TME targets in HGSOC, there is a pressing need for pre-clinical models that allow the assessment of new therapies, especially in combination. We set out to determine whether transplantable orthotopic models in immunocompetent mice would replicate some of the important molecular, cellular, and biomechanical features of the human diseased TMEs, as most previous studies have focused on the epithelial compartment. We had two advantages with these HGSOC models: first, we had previously conducted an extensive multi-level analysis of human HGSOC omental metastases (Pearce et al., 2018), and second, as we injected the murine cancer cells i.p., we were replicating the peritoneal dissemination that is thought to occur in human disease. We used polyclonal cell lines as we believe that they better reflect the intra-tumoral heterogeneity that is present in patients.

Although the murine cell lines only represented one group of HGSOC (i.e., those with dsDNA repair defects), there were significant correlations with pre-treatment human omental metastases and with primary tumors from the TCGA and ICGC datasets independent of whether the patient samples were DNA damage repair (DDR) deficient. The most commonly shared pathways related to the TME, especially in the matrisome and cell adhesion. This suggests that dsDNA repair defects are not major determinants of the TME, although *BRCA1* mutations are associated with higher T cell counts in patients (Clarke et al., 2009). Our tumor models recapitulate the CNAs that are the hallmark of HGSOC. The present study therefore highlights the importance of cell context and genomic content in mouse models—in other words, the correct genes in appropriate cell types give rise to tumors that recapitulate important components of the human TME. However, it is important to point out that 60577 and 30200 tumors were derived from the ovarian surface epithelium rather than the fallopian tube, although contemporary research strongly supports the fallopian tube origin (Karnezis et al., 2017). In addition, these tumors were syngeneic in FVB, whereas the HGS lines were syngeneic to B6.

Because we had extensively studied omental metastases in patients, we focused on murine omental metastases for many of our analyses, but there were significant correlations with primary tumors in all of the transcriptional data, suggesting that the tissue site does not necessarily influence the composition of the TME. This is also clear in studies of multiple human HGSOC biopsies from individual patients (Zhang et al., 2018).

One notable finding was the list of genes that were overexpressed in human and murine tumors compared to normal omentum, four of which we validated by IHC and flow cytometry. The top 40 genes are shown in Figure 2B, but there are 154 more that can be further studied, if confirmed at the protein level. Some of the commonly overexpressed genes were already known as therapeutic targets in ovarian and other cancers. For instance, folate receptor α is overexpressed by many human

(B) Heatmap of REACTOME DNA replication pathway genes across the murine tumors.

(C) Response of mice injected with 60577 or HGS2 to three cycles of chemotherapy (carboplatin 20 mg/kg, once per week). Survival curve and median survival are shown (n = 5 mice per group). The log rank p value is depicted on the survival curves. The start of the treatment is indicated by the red arrow.

(D) Heatmap of BIOCARTA IL-6 pathway genes across the murine tumors.

(E and F) Mice injected with 30200 (E) or HGS2 (F) were treated with isotype control or anti-IL-6 i.p. 2 mg/kg twice weekly starting 10 (30200) or 7 (HGS2) weeks after cell injection until endpoint. The log rank p value is depicted on the survival curves (for 30200, $n_{control} = 16$ and $n_{treated} = 11$; for HGS2, $n_{control} = 11$ and $n_{treated} = 12$). Analysis of the immune infiltrate was performed by flow cytometry on a different set of mice and Student's t test value is depicted on the bar plots. Each dot represents a tumor from an individual mouse. For 30200, two experiments pooled together are shown.

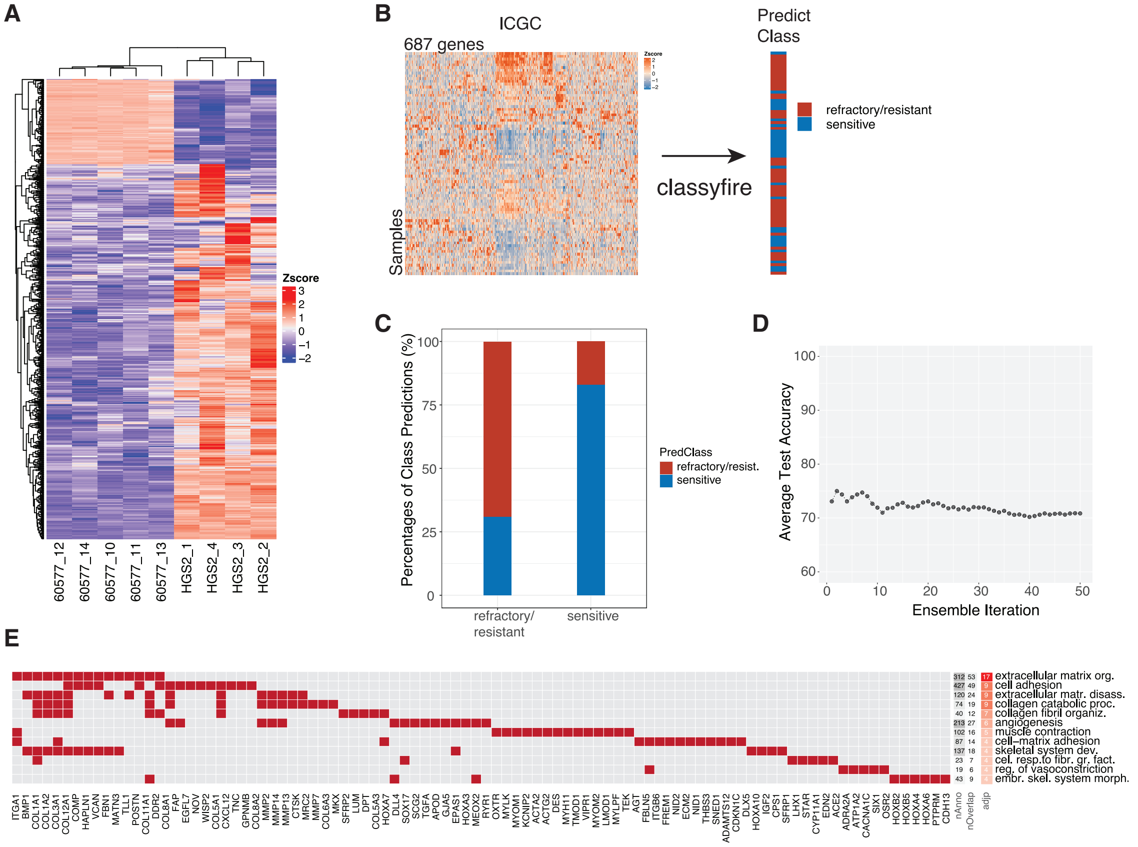

Figure 7. Multivariate Classification of Patient Response Based on Mouse Model Data

(A) Heatmap of 687 differentially expressed genes in 60577 (n = 5) versus HGS2 (n = 4) tumors (FDR < 0.0001, log fold change (FC) > |3|).

(B) Schematic of multivariate classification implemented with R package classyfire on ICGC data, using the 687 genes for the prediction of chemotherapy response. The 80 primary tumor samples of the ICGC dataset were used in this analysis.

(C) Bar plot illustrating the result of the classification ensemble accuracy on predicting class membership of previously unseen samples.

(D) Average test accuracy in relation to the number of support vector machine (SVM) ensembles used.

(E) Heatmap of the top GO biological processes enriched in the 687 genes (adjusted p < 0.05). adjp (adjusted p value), $-\log_{10}$; nAnno, number of genes in the gene set; nOverlap, number of overlapping genes between gene set and 687 gene list. Red squares denote genes in the top enriched GO processes. A maximum of 15 overlapping genes are shown for GO processes with nOverlap >15.

HGSOC cells and is now a target for antibody and chimeric antigen receptor (CAR) T cell therapies (Bergamini et al., 2016; Zhu et al., 2017). The vitamin D receptor is identified as a master transcription factor of stellate cells in PDAC (Sherman et al., 2014). Our previous work identified RUNX2 as a transcription factor that is common to a majority of the prognostic Matrix Index genes described earlier (Pearce et al., 2018). RUNX2 also plays important roles in the Hippo signaling, a pathway implicated in the regulation of tissue stiffness and signal transduction from the ECM as well as HGSOC progression (Passaniti et al., 2017). Another potential target, shared by the mouse and human tumor samples, is SLC34A2. Its protein product, NaPi2b, a type II sodium phosphate transporter, is highly expressed on the surface of ovarian and other malignant cells and is exploited in antibody-drug conjugates (Lin et al., 2015). The ability to delete genes of interest in our cell line-based tumor models will enable us and others to further understand the biology and therapeutic potential of individual targets.

There are also some notable differences between the mouse and human tumors that must be considered in translating preclinical studies. Probably the most important difference was higher numbers of TAMs and lower numbers of $CD3^+$ T cells, with a higher proportion of $CD4^+$ cells in the mouse versus human as this could influence immunotherapy response.

It is interesting that the common gene pathways that classified responses to chemotherapy in both human and mouse HGSOC were associated with cell adhesion and ECM and not DNA damage response or adaptive immune cell pathways. We can speculate that this means that less drug and/or fewer immune cells are able to reach malignant cell areas during chemotherapy, leading to a reduced response.

In conclusion, these orthotopic transplantable models may provide useful pre-clinical tools and help identify subgroups of HGSOC patients most likely to respond to specific therapies. The HGS cell lines are freely available, and all of the data associated with this paper is available at www.canbuild.org.uk.

STAR★METHODS

Detailed methods are provided in the online version of this paper and include the following:

- KEY RESOURCES TABLE
- LEAD CONTACT AND MATERIALS AVAILABILITY
- EXPERIMENTAL MODELS AND SUBJECT DETAILS
  - Study approval
  - Genetic mouse models
  - Generation and origin of murine cell lines
  - Orthotopic tumor growth
- METHOD DETAILS
  - Array Comparative Genomic Hybridization (aCGH)
  - RNA isolation and sequencing
  - Unsupervised clustering, differential expression and pathways analysis
  - Weighted gene co-expression network analysis (WGCNA)
  - Multivariate classification of ICGC platinum status using murine model data
  - Flow cytometry
  - Histopathology, immunohistochemistry and morphometry
  - Masson's trichrome
  - Generation of tissue-matrix heatmaps
  - Mechanical characterization
- QUANTIFICATION AND STATISTICAL ANALYSIS
- DATA AND CODE AVAILABILITY

SUPPLEMENTAL INFORMATION

Supplemental Information can be found online at https://doi.org/10.1016/j.celrep.2019.12.034.

ACKNOWLEDGMENTS

This project was funded by Cancer Research UK program grants A16354 and A25714 and ERC Advanced Grant ERC322566 (to F.R.B., C.B., G.G., P.K., A.L., R.M.D.-S., and O.M.T.P.); Cancer Research UK Career Establishment Award A27947 (to O.M.T.P.); Cancer Research UK core grant number A17196 (to O.J.S.); BBSRC CASE studentship BIDS3000032485 (to J.D.J.); Wellcome Trust Research Training Fellowship 20118/Z/16/Z (to O.H.); National Cancer Institute grant P50 CA228991; The Honorable Tina Brozman Foundation; the Dr. Miriam and Sheldon G. Adelson Medical Research Foundation; The Claneil Foundation; the Basser Center for BRCA (to R.D.); and the European Commission (PCIG13-GA-2013-618174) (to R.P.). This project has been funded in part with federal funds from the National Cancer Institute, National Institutes of Health, under contract HHSN261200800001E (to L.S.). The content of this publication does not necessarily reflect the views or policies of the US Department of Health and Human Services, nor does mention of trade names, commercial products, or organizations imply endorsement by the US government. We thank the surgeons from Barts Trust and the St. George's Hospital Trust and the Barts Cancer Institute (BCI) Tissue Bank for sample provision. We thank Richard Thompson, Dr. Alzbeta Talarovikova, and Dr Hagen Kulbe for their preliminary work in the breeding of GEMMs. This research was supported by the BCI/WHRI Flow Cytometry Core Facility. We also thank George Elia and the BCI Pathology Core for technical assistance. Most importantly, we express our gratitude to the patients for donating the samples, without which this work would not have been possible.

AUTHOR CONTRIBUTIONS

F.R.B., P.B., E.M., C.B., R.D., and O.J.S. designed the project; E.M. and P.B. designed and performed the bioinformatics analyses; C.B., G.G., O.H., P.K., A.L., J.M., C.P., R.M.D.-S., O.M.T.P., P.H., and J.D.J. designed, performed, and analyzed the mouse experiments and the analyses of mouse and human tissues; R.P., L.S., O.J.S., and R.D. made intellectual contributions; F.R.B. and E.M. wrote the manuscript; and all of the authors edited and commented on the manuscript.

DECLARATION OF INTERESTS

O.J.S. is supported by AstraZeneca, Novartis, and Cancer Research Technology. R.D. serves on the scientific advisory board of Repare Therapeutics and Siamab Therapeutics. F.R.B. serves on the scientific advisory board of Verseau Therapeutics. The authors declare no other competing interests.

Received: June 20, 2019
Revised: September 6, 2019
Accepted: December 10, 2019
Published: January 14, 2020

REFERENCES

Ahmed, A.A., Etemadmoghadam, D., Temple, J., Lynch, A.G., Riad, M., Sharma, R., Stewart, C., Fereday, S., Caldas, C., Defazio, A., et al. (2010). Driver mutations in TP53 are ubiquitous in high grade serous carcinoma of the ovary. J. Pathol. *221*, 49–56.

Anders, S., Pyl, P.T., and Huber, W. (2015). HTSeq–a Python framework to work with high-throughput sequencing data. Bioinformatics *31*, 166–169.

Bailey, P., Chang, D.K., Nones, K., Johns, A.L., Patch, A.M., Gingras, M.C., Miller, D.K., Christ, A.N., Bruxner, T.J., Quinn, M.C., et al.; Australian Pancreatic Cancer Genome Initiative (2016). Genomic analyses identify molecular subtypes of pancreatic cancer. Nature *531*, 47–52.

Bergamini, A., Ferrero, S., Leone Roberti Maggiore, U., Scala, C., Pella, F., Vellone, V.G., Petrone, M., Rabaiotti, E., Cioffi, R., Candiani, M., and Mangili, G. (2016). Folate receptor alpha antagonists in preclinical and early stage clinical development for the treatment of epithelial ovarian cancer. Expert Opin. Investig. Drugs *25*, 1405–1412.

Binnewies, M., Roberts, E.W., Kersten, K., Chan, V., Fearon, D.F., Merad, M., Coussens, L.M., Gabrilovich, D.I., Ostrand-Rosenberg, S., Hedrick, C.C., et al. (2018). Understanding the tumor immune microenvironment (TIME) for effective therapy. Nat. Med. *24*, 541–550.

Böhm, S., Montfort, A., Pearce, O.M., Topping, J., Chakravarty, P., Everitt, G.L., Clear, A., McDermott, J.R., Ennis, D., Dowe, T., et al. (2016). Neoadjuvant Chemotherapy Modulates the Immune Microenvironment in Metastases of Tubo-Ovarian High-Grade Serous Carcinoma. Clin. Cancer Res. *22*, 3025–3036.

Chatzimichali, E.A., and Bessant, C. (2016). Novel application of heuristic optimisation enables the creation and thorough evaluation of robust support vector machine ensembles for machine learning applications. Metabolomics *12*, 16.

Clarke, B., Tinker, A.V., Lee, C.H., Subramanian, S., van de Rijn, M., Turbin, D., Kalloger, S., Han, G., Ceballos, K., Cadungog, M.G., et al. (2009). Intraepithelial T cells and prognosis in ovarian carcinoma: novel associations with stage, tumor type, and BRCA1 loss. Mod. Pathol. *22*, 393–402.

Coward, J., Kulbe, H., Chakravarty, P., Leader, D., Vassileva, V., Leinster, D.A., Thompson, R., Schioppa, T., Nemeth, J., Vermeulen, J., et al. (2011). Interleukin-6 as a Therapeutic Target in Human Ovarian Cancer. Clin. Cancer Res. *17*, 6083–6096.

de Ronde, J.J., Klijn, C., Velds, A., Holstege, H., Reinders, M.J., Jonkers, J., and Wessels, L.F. (2010). KC-SMARTR: an R package for detection of statistically significant aberrations in multi-experiment aCGH data. BMC Res. Notes *3*, 298.

Delaine-Smith, R.M., Burney, S., Balkwill, F.R., and Knight, M.M. (2016). Experimental validation of a flat punch indentation methodology calibrated against unconfined compression tests for determination of soft tissue biomechanics. J. Mech. Behav. Biomed. Mater. *60*, 401–415.

Durinck, S., Spellman, P.T., Birney, E., and Huber, W. (2009). Mapping identifiers for the integration of genomic datasets with the R/Bioconductor package biomaRt. Nat. Protoc. *4*, 1184–1191.

Foster, D.S., Jones, R.E., Ransom, R.C., Longaker, M.T., and Norton, J.A. (2018). The evolving relationship of wound healing and tumor stroma. JCI Insight *3*, 99911.

Groszer, M., Erickson, R., Scripture-Adams, D.D., Lesche, R., Trumpp, A., Zack, J.A., Kornblum, H.I., Liu, X., and Wu, H. (2001). Negative regulation of neural stem/progenitor cell proliferation by the Pten tumor suppressor gene in vivo. Science *294*, 2186–2189.

Hansen, K.D., Irizarry, R.A., and Wu, Z. (2012). Removing technical variability in RNA-seq data using conditional quantile normalization. Biostatistics *13*, 204–216.

Hänzelmann, S., Castelo, R., and Guinney, J. (2013). GSVA: gene set variation analysis for microarray and RNA-seq data. BMC Bioinformatics *14*, 7.

Huang, D.W., Sherman, B.T., and Lempicki, R.A. (2009). Systematic and integrative analysis of large gene lists using DAVID bioinformatics resources. Nat. Protoc. *4*, 44–57.

Jia, D., Liu, Z., Deng, N., Tan, T.Z., Huang, R.Y., Taylor-Harding, B., Cheon, D.J., Lawrenson, K., Wiedemeyer, W.R., Walts, A.E., et al. (2016). A COL11A1-correlated pan-cancer gene signature of activated fibroblasts for the prioritization of therapeutic targets. Cancer Lett. *382*, 203–214.

Jonkers, J., Meuwissen, R., van der Gulden, H., Peterse, H., van der Valk, M., and Berns, A. (2001). Synergistic tumor suppressor activity of BRCA2 and p53 in a conditional mouse model for breast cancer. Nat. Genet. *29*, 418–425.

Karnezis, A.N., Cho, K.R., Gilks, C.B., Pearce, C.L., and Huntsman, D.G. (2017). The disparate origins of ovarian cancers: pathogenesis and prevention strategies. Nat. Rev. Cancer *17*, 65–74.

Langfelder, P., and Horvath, S. (2008). WGCNA: an R package for weighted correlation network analysis. BMC Bioinformatics *9*, 559.

Leong, H.S., Galletta, L., Etemadmoghadam, D., George, J., Köbel, M., Ramus, S.J., and Bowtell, D.; Australian Ovarian Cancer Study (2015). Efficient molecular subtype classification of high-grade serous ovarian cancer. J. Pathol. *236*, 272–277.

Lin, K., Rubinfeld, B., Zhang, C., Firestein, R., Harstad, E., Roth, L., Tsai, S.P., Schutten, M., Xu, K., Hristopoulos, M., and Polakis, P. (2015). Preclinical Development of an Anti-NaPi2b (SLC34A2) Antibody-Drug Conjugate as a Therapeutic for Non-Small Cell Lung and Ovarian Cancers. Clin. Cancer Res. *21*, 5139–5150.

Macintyre, G., Goranova, T.E., De Silva, D., Ennis, D., Piskorz, A.M., Eldridge, M., Sie, D., Lewsley, L.A., Hanif, A., Wilson, C., et al. (2018). Copy number signatures and mutational processes in ovarian carcinoma. Nat. Genet. *50*, 1262–1270.

Mantovani, A., Marchesi, F., Malesci, A., Laghi, L., and Allavena, P. (2017). Tumour-associated macrophages as treatment targets in oncology. Nat. Rev. Clin. Oncol. *14*, 399–416.

Martini, P., Sales, G., Massa, M.S., Chiogna, M., and Romualdi, C. (2013). Along signal paths: an empirical gene set approach exploiting pathway topology. Nucleic Acids Res *41*, e19.

Montfort, A., Pearce, O., Maniati, E., Vincent, B.G., Bixby, L., Böhm, S., Dowe, T., Wilkes, E.H., Chakravarty, P., Thompson, R., et al. (2017). A Strong B-cell Response Is Part of the Immune Landscape in Human High-Grade Serous Ovarian Metastases. Clin. Cancer Res. *23*, 250–262.

Newman, A.M., Liu, C.L., Green, M.R., Gentles, A.J., Feng, W., Xu, Y., Hoang, C.D., Diehn, M., and Alizadeh, A.A. (2015). Robust enumeration of cell subsets from tissue expression profiles. Nat. Methods *12*, 453–457.

Nieman, K.M., Kenny, H.A., Penicka, C.V., Ladanyi, A., Buell-Gutbrod, R., Zillhardt, M.R., Romero, I.L., Carey, M.S., Mills, G.B., Hotamisligil, G.S., et al. (2011). Adipocytes promote ovarian cancer metastasis and provide energy for rapid tumor growth. Nat. Med. *17*, 1498–1503.

Oza, A.M., Cook, A.D., Pfisterer, J., Embleton, A., Ledermann, J.A., Pujade-Lauraine, E., Kristensen, G., Carey, M.S., Beale, P., Cervantes, A., et al.; ICON7 trial investigators (2015). Standard chemotherapy with or without bevacizumab for women with newly diagnosed ovarian cancer (ICON7): overall survival results of a phase 3 randomised trial. Lancet Oncol. *16*, 928–936.

Passaniti, A., Brusgard, J.L., Qiao, Y., Sudol, M., and Finch-Edmondson, M. (2017). Roles of RUNX in Hippo Pathway Signaling. Adv. Exp. Med. Biol. *962*, 435–448.

Patch, A.M., Christie, E.L., Etemadmoghadam, D., Garsed, D.W., George, J., Fereday, S., Nones, K., Cowin, P., Alsop, K., Bailey, P.J., et al.; Australian Ovarian Cancer Study Group (2015). Whole-genome characterization of chemoresistant ovarian cancer. Nature *521*, 489–494.

Pearce, O.M.T., Delaine-Smith, R.M., Maniati, E., Nichols, S., Wang, J., Böhm, S., Rajeeve, V., Ullah, D., Chakravarty, P., Jones, R.R., et al. (2018). Deconstruction of a Metastatic Tumor Microenvironment Reveals a Common Matrix Response in Human Cancers. Cancer Discov. *8*, 304–319.

Perets, R., Wyant, G.A., Muto, K.W., Bijron, J.G., Poole, B.B., Chin, K.T., Chen, J.Y., Ohman, A.W., Stepule, C.D., Kwak, S., et al. (2013). Transformation of the fallopian tube secretory epithelium leads to high-grade serous ovarian cancer in Brca;Tp53;Pten models. Cancer Cell *24*, 751–765.

Ritchie, M.E., Phipson, B., Wu, D., Hu, Y., Law, C.W., Shi, W., and Smyth, G.K. (2015). limma powers differential expression analyses for RNA-sequencing and microarray studies. Nucleic Acids Res. *43*, e47.

Robinson, M.D., McCarthy, D.J., and Smyth, G.K. (2010). edgeR: a Bioconductor package for differential expression analysis of digital gene expression data. Bioinformatics *26*, 139–140.

Ruifrok, A.C., and Johnston, D.A. (2001). Quantification of histochemical staining by color deconvolution. Anal. Quant. Cytol. Histol. *23*, 291–299.

Schneider, C.A., Rasband, W.S., and Eliceiri, K.W. (2012). NIH Image to ImageJ: 25 years of image analysis. Nat. Methods *9*, 671–675.

Schwender, H. (2019). siggenes: Multiple Testing using SAM and Efron's Empirical Bayes Approaches. R package version 1.60.0 (R Foundation for Statistical Computing).

Seshan, V.E., and Olshen, A. (2019). DNAcopy: DNA copy number data analysis. R package version 1.60.0 (R Foundation for Statistical Computing).

Sherman, M.H., Yu, R.T., Engle, D.D., Ding, N., Atkins, A.R., Tiriac, H., Collisson, E.A., Connor, F., Van Dyke, T., Kozlov, S., et al. (2014). Vitamin D receptor-mediated stromal reprogramming suppresses pancreatitis and enhances pancreatic cancer therapy. Cell *159*, 80–93.

Socovich, A.M., and Naba, A. (2018). The cancer matrisome: from comprehensive characterization to biomarker discovery. Semin. Cell Dev. Biol *89*, 157–166.

Stone, R.L., Nick, A.M., McNeish, I.A., Balkwill, F., Han, H.D., Bottsford-Miller, J., Rupairmoole, R., Armaiz-Pena, G.N., Pecot, C.V., Coward, J., et al. (2012). Paraneoplastic thrombocytosis in ovarian cancer. N. Engl. J. Med. *366*, 610–618.

Stuckelberger, S., and Drapkin, R. (2018). Precious GEMMs: emergence of faithful models for ovarian cancer research. J. Pathol. *245*, 129–131.

Szabova, L., Bupp, S., Kamal, M., Householder, D.B., Hernandez, L., Schlomer, J.J., Baran, M.L., Yi, M., Stephens, R.M., Annunziata, C.M., et al. (2014). Pathway-specific engineered mouse allograft models functionally recapitulate human serous epithelial ovarian cancer. PLoS One *9*, e95649.

The Cancer Genome Atlas Research Network (2011). Integrated genomic analyses of ovarian carcinoma. Nature *474*, 609–615.

Walton, J., Blagih, J., Ennis, D., Leung, E., Dowson, S., Farquharson, M., Tookman, L.A., Orange, C., Athineos, D., Mason, S., et al. (2016). CRISPR/Cas9-Mediated Trp53 and Brca2 Knockout to Generate Improved Murine Models of Ovarian High-Grade Serous Carcinoma. Cancer Res. *76*, 6118–6129.

Zhai, Y., Wu, R., Kuick, R., Sessine, M.S., Schulman, S., Green, M., Fearon, E.R., and Cho, K.R. (2017). High-grade serous carcinomas arise in the mouse oviduct via defects linked to the human disease. J. Pathol. *243*, 16–25.

Zhang, A.W., McPherson, A., Milne, K., Kroeger, D.R., Hamilton, P.T., Miranda, A., Funnell, T., Little, N., de Souza, C.P.E., Laan, S., et al. (2018). Interfaces of Malignant and Immunologic Clonal Dynamics in Ovarian Cancer. Cell *173*, 1755–1769.e22.

Zhu, X., Cai, H., Zhao, L., Ning, L., and Lang, J. (2017). CAR-T cell therapy in ovarian cancer: from the bench to the bedside. Oncotarget *8*, 64607–64621.

STAR★METHODS

KEY RESOURCES TABLE

REAGENT or RESOURCE	SOURCE	IDENTIFIER
Antibodies		
Rat anti-mouse CD45-BV785 (Clone 30-F11)	Biolegend	Cat# 103149; RRID:AB_2564590
Armenian hamster anti-mouse CD3-PE-Cy7 (Clone 145-2C11)	Biolegend	Cat# 100320; RRID:AB_312685
Rat anti mouse CD4-BV605	Biolegend	Cat#100548; RRID:AB_2563054
Rat anti-mouse CD8a-APC (Clone 53-6.7)	eBioscience	Cat# 17-0081-83; RRID:AB_469336)
Rat anti-mouse/human CD11b-BV650 (Clone M1/70)	Biolegend	Cat# 101239; RRID:AB_11125575)
Armenian hamster anti-mouse CD11c-FITC, (Clone N418)	eBioscience	Cat#11-0114-82; RRID:AB_464940
Rat anti-mouse F4/80-PE (Clone BM8)	Biolegend	Cat#123110; RRID:AB_893486
Rat anti-mouse Ly6C -eFluor450 (Clone HK1.4)	eBioscience	Cat#48-5932-82; RRID:AB_10805519
Rat anti-mouse Ly6G(Gr1)-AF700 (Clone RB6-BC5)	eBioscience	Cat#56-5931-82; RRID:AB_10805519
Rat anti-mouse CD19 PerCP-Cy5.5 (Clone 6D5)	eBioscience	Cat#115534; RRID:AB_2072925
Rat anti-mouse I-A/I-E- APC-Cy7 (Clone M5/114)	Biolegend	Cat#107628; RRID:AB_2069377
InVivoMAb anti-mouse IL-6	BioXCell	Cat#BE0046; RRID:AB_1107709
InVivoMAb rat IgG1 isotype control, anti-horseradish peroxidase	BioXCell	Cat# BE0088; RRID:AB_1107775
F(ab')2-Goat anti-Rat IgG (H+L) Highly Cross-Adsorbed Secondary HRP	Thermo Fisher	Cat# A24555; RRID:AB_2536023
Goat anti-rabbit biotinylated IgG	Vector Laboratories	Cat# BA-1000; RRID:AB_2313606
Anti-rat IgG	Vector Laboratories	Cat# BA-4001; RRID:AB_10015300
Anti-mouse IgG	Vector Laboratories	Cat# BA-2000; RRID:AB_2313581
Rat monoclonal anti-CD3	Abcam	Cat# ab11089; RRID: AB_369097
Rabbit polyclonal anti-human CD3	Agilent	Cat# A0452; RRID: AB_2335677
Rat monoclonal anti-F4/80	Serotec/BioRad	Cat# MCA497; RRID:AB_2098196
Rat monoclonal anti-endomucin (V.7C7)	Santa Cruz Biotechnology	Cat# sc-65495; RRID:AB_2100037
Mouse monoclonal anti-PECAM-1 (0.N.100)	Santa Cruz Biotechnology	Cat# sc-71872; RRID:AB_1125653)
Rabbit polyclonal anti-actin, smooth muscle	Abcam	Cat# ab5694; RRID:AB_2223021
Mouse monoclonal anti-actin, smooth muscle Clone 1A4	Sigma-Aldrich	Cat# A2547; RRID:AB_476701
Rabbit monoclonal anti-Ki67 [SP6]	Abcam	Cat#ab16667; RRID:AB_302459)
Rabbit polyclonal anti-VCAN	Sigma-Aldrich	Cat# HPA004726; RRID:AB_1080561)
Rabbit polyclonal anti-COL11A1	Sigma-Aldrich	Cat#HPA052246; RRID:N/A
Rabbit polyclonal anti-COL11A1	Novus	Cat# NBP2-58159; RRID:N/A
Rabbit polyclonal anti-COL1A1	Abcam	Cat# ab21286; RRID:AB_446161)
Rabbit polyclonal anti-FN1	Sigma-Aldrich	Cat# F3648; RRID:AB_476976
Rabbit polyclonal anti-CTSB	Novus	Cat# NBP1-19797; RRID:AB_2086951
Rabbit polyclonal anti-COMP	GeneTex	Cat# GTX14515; RRID:AB_845475)
Rabbit monoclonal anti-Runx2 [EPR14334]	Abcam	Cat# ab192256; RRID:AB_2713945
Rabbit monoclonal anti-Vitamin D3Receptor (D2K6W)	Cell Signaling Technology	Cat#12550; RRID:AB_2637002
Rabbit monoclonal anti-Folate Binding Protein EPR20277	Abcam	Cat# ab221543; RRID: N/A
TruStain fcX (anti-mouse CD16/32) Antibody	Biolegend	Cat#101320; RRID:AB_1574975

(Continued on next page)

Continued		
REAGENT or RESOURCE	SOURCE	IDENTIFIER
Biological Samples		
Human HGSOC omental metastasis samples	Barts Health NHS Trust, St. George's University Hospitals NHS Foundation Trust and Barts Gynae Tissue Bank	(https://directory.biobankinguk.org/Profile/Biobank/GBR-1-128) HTA license number 12199 (REC no: 10/H0304/14 and 15/EE/0151)
Chemicals, Peptides, and Recombinant Proteins		
collagenase from Clostridium histolyticum	Sigma-Aldrich	Cat# C9263
DNase I from bovine pancreas	Sigma-Aldrich	Cat# D4513
HBSS (10X), no calcium, no magnesium, no phenol red	GIBCO	Cat# 14185-045
Fixable Viability Dye eFluor® 506	eBioscience	Cat# v65-0866-18
Formalin solution neutral buffered 10%	Sigma Aldrich	Cat# HT501128
Xylene, Technical, Fisher Chemical	Fisher Scientific	Cat# X/0100/17
Ethanol, 99.8%, as ethanol,anhydrous,(denat. with 2% IPA + 2% MEK)	Fisher Scientific	Cat#12367103
Hydrogen Peroxide 30-32% (w/w) (100 Volumes), Certified AR for Analysis	Fisher Scientific	Cat# H/1800/15
Methanol 99.9% (GLC) 0.7915 g/mL for analysis CertiFied AR	Fisher Scientific	Cat# M/4000/PB17
Antibody diluent	Zytomed Systems	Cat# ZUC025-100
Diaminobenzidine substrate-chromogen (Dako Liquid DAB+ Substrate Chromogen System)	Dako	Cat# K3468
Gill's hematoxylin I	Sigma-Aldrich	Cat# GHS1128
DPX Mountant for histology	Sigma-Aldrich	Cat# 06522
Bouin's Solution	Sigma Aldrich	Cat# HT10132
Weigert's Iron Hematoxylin Solution	Sigma Aldrich	Cat# HT1079
Antigen Retrieval Buffer (100X Tris-EDTA Buffer, pH 9.0)	Abcam	Cat# ab93684
Novocastra protein block	Leica Biosystem	Cat# RE7102-CE
Bovine Serum Albumin	Sigma Aldrich	Cat# A4503
Goat serum	Sigma Aldrich	Cat# G9023
Phosphate-buffered saline (PBS) (1x)	GIBCO	Cat# 141-90-094
Proteinase K in TE-CaCl2 buffer pH8	Invitrogen	Cat# 25530015
Tween 20	Sigma-Aldrich	Cat# P7949-500ml
Antigen Unmasking Solution, Citric Acid Based	Vector Laboratories	Cat# H3300
Triton X-100	Sigma-Aldrich	Cat#T8787
Protein Block, Serum-Free	DAKO Agilent	Cat# X0909
EDTA 0.5M	Thermo fisher	Cat# AM9262
Doxycycline hyclate	Sigma-Aldrich	Cat# D9891
DMEM:F12 1:1 with GlutaMax	GIBCO	Cat# 31331-028
Fetal Bovine Serum	Hyclone	Cat# 11521831
Penicillin-Streptomycin (10,000 U/mL)	Invitrogen	Cat# 15140-122
Insulin, Transferrin, Selenium, Sodium Pyruvate Solution (ITS-A)	Invitrogen	Cat# 51300
Hydrocortisone, γ-irradiated, powder	Sigma	Cat# H0135
Murine Epidermal Growth Factor (EGF)	Sigma	Cat# E4127
Antibiotic-Antimycotic (100X)	GIBCO	Cat# 5240-062
0.5% Trypsin-EDTA (10X)	GIBCO	Cat#15400-054
Carboplatin	Hospira	Cas#41575-94-4

(*Continued on next page*)

Continued

REAGENT or RESOURCE	SOURCE	IDENTIFIER
Fixable Viability Dye eFluor506	eBioscience	Cat# 65-0866-14
Collagenase type I	Fisher Scientific	Cat# 17018-029
Glacial acetic acid	Fisher Scientific	Cat# 10021123
Critical Commercial Assays		
Trichrome Stain (Masson) Kit	Sigma-Aldrich	Cat# HT15-1KT
anti-rat ImmPRESS-HRP reagent (MP7444, Vector	Vector Laboratories	Cat# MP7444
ImmPress HRP anti-rabbit (MP7451, Vector)	Vector Laboratories	Cat# MP7451
Super Sensitive Polymer HRP IHC	BioGenex	Cat# QD430-XAKE
RNAlater-ice	Thermo Fisher Scientific	Cat# 4427575
RNeasy Mini kit	Quiagen	Cat# 74104
RNA NanoChips	Agilent	Cat# 5067-1511
QiaShredders	Quiagen	Cat# 79654
RNeasy Plus Mini kit	Quiagen	Cat# 74134
DNeasy Blood and Tissue kit	Quiagen	Cat# 69504
Deposited Data		
Murine and human omental metastasis data	GEO	GSE132289
Experimental Models: Cell Lines		
60577	Laboratory of Difilippantonio S.	Szabova et al., 2014
30200	Laboratory of Difilippantonio S.	Szabova et al., 2014
HGS1	This paper	N/A
HGS2	This paper	N/A
HGS3	This paper	N/A
HGS4	This paper	N/A
Experimental Models: Organisms/Strains		
PAX8-rtTA, TetO-Cre mice	Laboratory of Ronny Drapkin	Perets et al., 2013
TP53fl/fl [B6.129P2-Trp53tm1Brn/J] mice	Jackson Laboratory	N/A
PTENfl/fl mice	Laboratory of Bart Vanhaesebroeck	Groszer et al., 2001
BrCa2fl/fl mice	Laboratory of Ashok Venkitaraman	Jonkers et al., 2001
FVB mice	Charles River	N/A
C57/Bl6J mice	Charles River	N/A
Software and Algorithms		
FlowJo software, Tree Star, Inc.	FlowJo Software for Mac Version *10*.Ashland	https://www.flowjo.com/solutions/flowjo
NPD.view 2.7.25 software		https://www.hamamatsu.com/eu/en/product/type/U12388-01/index.html
ImageJ 1.48v image processing and analysis program (NIH, Bethesda, MD) with color threshold and color deconvolution plug-ins	Schneider et al., 2012; Ruifrok and Johnston, 2001	https://imagej.nih.gov/ij/ and https://imagej.net/Colour_Deconvolution
Definiens® software	(Definiens AG, Germany)	http://www.astrazeneca.com
Graphpad Prism, San Diego, CA	NA	https://www.graphpad.com
R v3.5.1	NA	http://www.R-project.org
KCsmart	Bioconductor	de Ronde et al., 2010
DNACopy	Bioconductor	Seshan and Olshen, 2019
HTSeq	https://htseq.readthedocs.io/en/release_0.11.1/	Anders et al., 2015
EdgeR	Bioconductor	Robinson et al., 2010
limma	Bioconductor	Ritchie et al., 2015
DAVID v6.8	https://david.ncifcrf.gov	Huang et al., 2009
Clipper	Bioconductor	Martini et al., 2013

(*Continued on next page*)

Continued

REAGENT or RESOURCE	SOURCE	IDENTIFIER
GSVA	Bioconductor	Hänzelmann et al., 2013
siggenes	Bioconductor	Schwender, 2019
biomaRt	Bioconductor	Durinck et al., 2009
factoextra	CRAN	https://cran.r-project.org/web/packages/factoextra/index.html
dnet	CRAN	https://cran.r-project.org/web/packages/dnet/index.html
ggplot2	CRAN	https://cran.r-project.org/web/packages/ggplot2/index.html
WGCNA	CRAN	Langfelder and Horvath, 2008
classyfire	CRAN	Chatzimichali and Bessant, 2016
Other		
BD LSR Fortessa cytometer	BD	N/A
Hamamatsu NanoZoomer S210 Slide Scanner	N/A	N/A
3DHISTECH Panoramic 250 digital slide scanner	3DHISTECH, Hungary	N/A
70 μm strainers	Fisher Scientific	N/A
GentleMACS M Tubes	Miltenyi	Cat# 130-093-236
Primaria flasks	Corning	Cat# 353808
Instron ElectroPuls E1000	Instron, UK	N/A

LEAD CONTACT AND MATERIALS AVAILABILITY

This study did not generate new unique reagents. Further information and requests for resources and reagents should be directed to and will be fulfilled by the Lead Contact, Frances Balkwill (f.balkwill@qmul.ac.uk).

EXPERIMENTAL MODELS AND SUBJECT DETAILS

Study approval

Murine models

All experimental procedures observed the guidelines approved by the ethics committees of QMUL under the Home Office Project license PBE3719B3. For survival experiments, mice were culled when they reached humane endpoint as defined in the license.

Patient samples

Samples were kindly donated by HGSOC patients undergoing surgery at Barts Health NHS Trust and St. George's University Hospitals NHS Foundation Trust. Tissues deemed by a pathologist to be surplus to diagnostic and therapeutic requirements were collected along with clinical data under the Barts Gynae Tissue Bank HTA license number 12199 (REC no: 10/H0304/14 and 15/EE/0151). Patients gave written informed consent and the study was approved by a UK national review board. Studies were conducted in accordance with the Declaration of Helsinki and the International Ethical Guidelines for Biomedical Research Involving Human Subjects.

Genetic mouse models

Trp53$^{fl/fl}$ mice were acquired from the Jackson Laboratory [B6.129P2-*Trp53*^{tm1Brn}/J]. *Pten*$^{fl/fl}$ mice (Groszer et al., 2001) were a kind gift from Bart Vanhaesebroeck (UCL Cancer Institute, University College London, UK). *Brca2*$^{fl/fl}$ mice were a kind gift from Ashok Venkitaraman (MRC Cancer Unit, University of Cambridge, UK) as originally described in Jonkers et al. (2001). Mice were genotyped by TransnetYX (Cordova, USA). *Trp53*$^{fl/fl}$;*Pten*$^{fl/fl}$ mice were received fully-backcrossed onto a C57BL/6J background. *Brca2*$^{fl/fl}$ mice were backcrossed in house for ten generations. When the cell lines were generated, the strain background was checked by SNP profiling and corresponded to 97% C57BL/6J background. In order to induce the expression of Cre, mice were treated with 0.2 mg/ml Doxycycline hyclate (Sigma) in drinking water for 14 days after weaning. All experiments were conducted with female mice.

Generation and origin of murine cell lines

HGS lines were derived from individual tumors collected in cold PBS, minced with a scalpel, and incubated with 1 mg/ml collagenase type I (Fisher, 17018-029). After being split once onto collagen-coated plates, cells were transferred into Primaria flasks (Corning) and then propagated as polyclonal lines on normal cell-culture plastic (Corning). Complete medium was DMEM:F12 1:1 with GlutaMax

(GIBCO) with the addition of 4% FBS (Hyclone), 1x Pen/Strep (Invitrogen), 1x insulin/transferrin/selenium (Invitrogen, 51300), 100 ng/ml hydrocortisone (Sigma, H0135), 20 ng/ml murine Epidermal Growth Factor (EGF, Sigma, E4127), 1x antibiotic-antimycotic (GIBCO). Cells were trypsinized with 0.05% trypsin-EDTA (GIBCO). 60577 and 30200 cell lines were derived and cultured as described (Szabova et al., 2014).

Orthotopic tumor growth

Cell lines were trypsinized, washed in medium, and resuspended in PBS to 10×10^6 cells in 300 μl injected i.p. into 8-week old FVB mice (60577, 30200) or 6-7-week old C57BL/6J mice (HGS1-4) from Charles River, UK. Mice were treated with 2 mg/kg anti-IL-6 or isotype control (BioXCell) twice weekly i.p., starting three weeks (60577 model), ten weeks (30200 model) or seven weeks (HGS2 model) after cell injection, until the end of the experiment. Carboplatin (20 mg/kg) was administered i.p. once a week for three weeks starting at week 3 (60577 model), or week 8 (HGS2 model).

METHOD DETAILS

Array Comparative Genomic Hybridization (aCGH)

Mouse genomic DNA was isolated from frozen tumors, cell lines or mouse tails (for reference controls) using DNeasy Blood and Tissue kit (QIAGEN). Frozen tissue was processed using TissueLyser II homogenizer at 15 Hz for 40 s prior to DNA extraction. aCGH was performed using SurePrint G3 mouse CGH 1M microarray kit (Agilent Technologies) or Cancer Research human CGH+SNP array (Agilent Technologies) as described (Perets et al., 2013). Probe signal intensities were obtained using the feature extraction software analysis provided by Agilent. Significant recurrent CNA were identified using R package KCsmart (de Ronde et al., 2010), applying the function findSigLevelTrad with 1000 permutations and a threshold at $p < 0.05$. To identify CNAs in individual cell lines and tumors we used the R package DNACopy to generate segmentation data for each sample (Seshan and Olshen, 2019). Non-redundant copy number regions were then identified from the segmentation data and medoids representing a copy number profile for a given genomic region generated using the *CNregions* function from the R package iClusterPlus.

RNA isolation and sequencing

Total RNA was extracted from frozen murine tumors and non-diseased omenta. The samples were transferred into RNAlater and homogenized with Miltenyi GentleMACS in RLT buffer and further processed using QIAGEN RNeasy Mini kit with on-column DNase digestion. RNA integrity numbers (RIN) were above 7.0 with the exception of GEMM tumors which were more degraded. For human samples total RNA was extracted from nine omental tumors and three non-involved omental samples. The samples were prepared as described (Pearce et al., 2018) using QiaShredders and the QIAGEN RNeasy Plus Mini kit with on-column DNase digestion. RNA quality was analyzed on the Agilent Bioanalyzer 2100 using RNA Nano Chips. RIN numbers were above 7.0. Library preparation and RNASeq were performed by the Wellcome Trust Centre (Oxford, UK) using RiboZero to deplete rRNA species. Sequencing was performed to ~40x mean depth on the Illumina HiSeq4000 platform, strand-specific, generating 150 bp paired-end reads. For murine samples, RNASeq reads were mapped to the mouse genome (mm10, Genome Reference Consortium GRCm38) and for human samples to the human genome (hg19, Genome Reference Consortium GRCh37) in strand-specific mode. Number of reads aligned to the exonic region of each gene were counted using htseq-count (Anders et al., 2015) based on the Ensembl annotation. Only genes that achieved at least one read count per million reads (cpm) in at least twenty-five percent of the samples were kept. Conditional quantile normalization (Hansen et al., 2012) was performed accounting for gene length and GC content and a $\log_2$-transformed RPKM expression matrix was generated. Four tumor samples and six non-diseased omenta from our prior RNaseq dataset (GSE71340) were included in the human analysis giving a total of nine tumors and nine non-diseased omenta.

Unsupervised clustering, differential expression and pathways analysis

Principal component and hierarchical cluster analysis were performed on the transformed RPKM matrix using R package factoextra. Differential expression analysis was performed in Edge R using limma (Ritchie et al., 2015; Robinson et al., 2010). False discovery rate (FDR) was calculated using the Benjamini-Hochberg method. Over-representation analysis for GO Biological process, KEGG and REACTOME pathways on the common tumor-associated differentially expressed genes in murine models (Figure 1C) was performed using DAVID v6.8 https://david.ncifcrf.gov. The R package clipper (v3.8) was used to implement topological gene set analysis in mouse and human omental tumors versus non-diseased omentum (Figure 2C). Heatmaps illustrate concordant orthologous genes in human and mouse models in commonly enriched gene sets (pathway threshold $p < 0.05$). Gene Ontology Biological process enrichment analysis was performed using the R package dnet and dotplot (Figure 2D) was constructed using ggplot2. Single sample gene-set enrichment analysis (Figure 6A) which calculates a gene-set enrichment score per sample was performed using the R package GSVA (Hänzelmann et al., 2013). To identify pathways differentially enriched between mouse models we used the function sam from R package siggenes on the ssGSEA enrichment scores (Schwender, 2019).

Weighted gene co-expression network analysis (WGCNA)

WGCNA (Langfelder and Horvath, 2008) was used to identify correlation patterns among genes on the normalized and transformed RSEM matrix of the ICGC OV-AU dataset (Patch et al., 2015). The module eigengene was used as a measure of module expression.

To relate to C1-C5 molecular subtypes, gene set GSVA enrichment scores were used as sample traits and correlated to module eigen genes. To determine the enrichment of differentially expressed mouse genes in modules generated by WGCNA, mouse identifiers were first mapped to their corresponding human HGCN Symbol using the R package biomaRt. Module gene enrichment was then determined using the function userListEnrichment in the WGCNA package and by gene expression/gene module correlations. Using the ICGC OV-AU set and the TCGA ovarian Affymetrix U133a 2.0 Array (The Cancer Genome Atlas Research Network, 2011) we also constructed consensus modules by WGCNA and related module eigen genes to survival, C1-C5 molecular subtypes and the differentially expressed mouse genes as described above.

Multivariate classification of ICGC platinum status using murine model data

The top differentially expressed genes in 60577 versus HGS2 (FDR < 0.0001 and logFC $> |3|$) with human orthologs sufficiently detected in ICGC were obtained, a total of 687 genes. Using the expression of these 687 genes multivariate classification was applied on the 80 primary tumor samples of the ICGC data to predict patients' platinum response status. This was performed using the R package classyfire (Chatzimichali and Bessant, 2016), which implements ensemble support vector machine (SVM) training and rigorous performance evaluation. The cfBuild function, designed to produce an ensemble of classifiers (with each classifier being a collection of multiple individually trained SVMs) was used with bootNum set to 100 and ensNum set to 50. To determine statistical significance the cfPermute function was used with PermNum set to 100, bootNum 10 and ensNum 20.

Flow cytometry

Mouse tumors were minced and incubated in collagenase from Clostridium histolyticum (Sigma), 2 mg/ml, and DNase I from bovine pancreas, 25 μg/ml (Sigma) in HBSS (Sigma) for 20-30 min at 37°C. The lysate was strained through 70 μm strainers (Fisher Scientific). Cells were counted and 0.5-1x10^6 cells stained in PBS, 2.5% BSA, 2 mM EDTA after blocking with Trustain (Biolegend) for 15 min. Staining antibodies were diluted 1:200 unless differently specified: anti-CD45-BV785 (1:100, Biolegend), anti-CD3 PE-Cy7 1:50 (Biolegend), anti-CD4 BV605 1:100 (Biolegend), anti-CD8 APC (eBioscience), anti-CD11b BV650 (Biolegend), anti-CD11c FITC (eBioscience), anti-F4/80 PE (Biolegend), anti-Ly6C eFluor450 (eBioscience, 1:100), anti-Ly6G(Gr1) AF700 (eBioscience), anti-CD19 PerCP-Cy5.5 (eBioscience), anti-MHCII APC-Cy7 (Biolegend). Viability was assessed with Fixable Viability Dye eFluor506 (eBioscience) diluted 1:500. Staining was performed for 30 min at 4°C. The cells were finally washed, fixed and analyzed on a BD LSR Fortessa cytometer. Appropriate Fluorescence Minus One (FMO) controls were used in these experiments. Analysis was performed with the FlowJo software. Human omental tissue was processed and stained similarly as previously described (Böhm et al., 2016).

Histopathology, immunohistochemistry and morphometry

Omental tumors were dissected, fixed in 4% formaldehyde for 24 hr, paraffin-embedded and sectioned (4 μm), followed by H&E staining. Formalin-fixed paraffin-embedded sections (4 μm) of omental samples were heated for 1hr at 60°C and then submerged twice in xylene for 5min. Slides were then gradually re-hydrated by submerging for 2min in each of the following ethanol solutions: 100%, 90%, 70%, 50% and finally in ddH_2O for 3min. Antigen retrieval was performed for all stainings apart from alpha-SMA, FN1 and COMP. The slides were subsequently washed, treated with 3% H_2O_2 (Fisher Scientific, H/1800/15) in PBS, methanol or water for 5-10min, washed again and blocked with blocking buffer for 20min-1hr. The primary antibody was added in in blocking buffer and incubated at ambient temperature (Table S7). Slides were washed 3 times and the primary antibody was detected as described in Table S7. Color was developed with Diaminobenzidine substrate-chromogen (Dako Liquid DAB+ Substrate Chromogen System, K3468 Dako) and tissues were counterstained with Gill's hematoxylin I (Sigma-Aldrich, GHS1128), washed, dehydrated in ethanol and mounted in DPX (Sigma-Aldrich, 06522).

Masson's trichrome

For Masson's trichrome staining of omental samples, the Trichrome Stain (Masson) Kit (HT15-1KT) from Sigma was used. Briefly, formalin-fixed paraffin-embedded 4-μm sections were submerged twice in xylene for 5min and gradually re-hydrated by submerging for 2min in each of the following ethanol solutions: 100% (X2), 90%, 70%, and finally 50%. Slides were further hydrated for 3min in ddH2O and fixed in preheated Bouin's for one hour at 60°C. Upon fixation, sections were washed in running water until the yellow color disappeared, rinsed in two changes of distilled water and stained in working Weigert's Iron Hematoxylin Solution for 5 minutes (Sigma, HT1079-1SET). Subsequently, sections were washed in running tap water for 2 minutes, rinsed in ddH_2O and stained in Biebrich Scarlet-Acid Fucshin for 15 minutes. Sections were then rinsed in ddH_2O and immersed in Working Phosphotungstic/Phosphomolybdic Acid solution for 10-15 minutes, until collagen fibers were not red. Next, Aniline Blue Solution was applied for 30 minutes; sections were rinsed in ddH_2O and placed in 1% acetic acid for 3 minutes. Finally, sections were dehydrated very quickly in two changes of 90% alcohol, followed by 2 changes of absolute alcohol, cleared in xylene and mounted.

Generation of tissue-matrix heatmaps

Serial sections of the HGS mouse tumors, along with normal omenta were stained by immunohistochemistry for all 6 ECM molecules and scanned with Hamamatsu NanoZoomer S210 Slide Scanner. Digital images from the serial sections were obtained using the NPD.view 2.7.25 software under the same magnification. The ImageJ (ImageJ 1.48v) image processing and analysis program (NIH, Bethesda, MD) with color threshold and color deconvolution plug-ins was used for the generation of tissue matrisome

heatmaps. Briefly, images were aligned and color deconvoluted and all six isolated DAB images were subsequently converted to single color images and compiled to a Z stack (AVG stack), selecting average intensity. A tissue matrix heatmap was generated by applying an edited Royal filter to the AVG stack, so that each color corresponds to either the absence (black) or the presence *in situ* of one (dark blue), two (cyan), three (green), four (yellow), five (orange) or six (red) ECM molecules. Finally, a Calibration bar was applied to the AVG stack with all seven colors and the heatmap image was flattened before export.

Mechanical characterization

Mechanical characterization was performed on frozen murine or human tissues using a flat-punch indentation methodology on an Instron ElectroPuls E1000 (Instron, UK) equipped with a 10 N load cell (resolution = 0.1 mN) as described (Delaine-Smith et al., 2016; Pearce et al., 2018).

QUANTIFICATION AND STATISTICAL ANALYSIS

Tissue sections were scanned either using a 3DHISTECH Panoramic 250 digital slide scanner (3DHISTECH, Hungary) or with Hamamatsu NanoZoomer S210 Slide-Scanner and the scans analyzed using Definiens® software (Definiens AG, Germany). Bioinformatic analyses were performed in the statistical programming language R (version 3.5.1). All other statistical analyses were performed using Graphpad Prism, San Diego, CA. Statistical tests used, n numbers and P values are displayed in the appropriate figures and figure legends. P values < 0.05 were considered statistically significant.

DATA AND CODE AVAILABILITY

The accession number for the RNASeq data reported in this paper is Gene Expression Omnibus (GEO): GSE132289. R markdown scripts enabling the main steps of the analysis are available from the Lead Contact upon reasonable request.

Cell Reports, Volume *30*

Supplemental Information

Mouse Ovarian Cancer Models Recapitulate the Human Tumor Microenvironment and Patient Response to Treatment

Eleni Maniati, Chiara Berlato, Ganga Gopinathan, Owen Heath, Panoraia Kotantaki, Anissa Lakhani, Jacqueline McDermott, Colin Pegrum, Robin M. Delaine-Smith, Oliver M.T. Pearce, Priyanka Hirani, Joash D. Joy, Ludmila Szabova, Ruth Perets, Owen J. Sansom, Ronny Drapkin, Peter Bailey, and Frances R. Balkwill

Figure S1

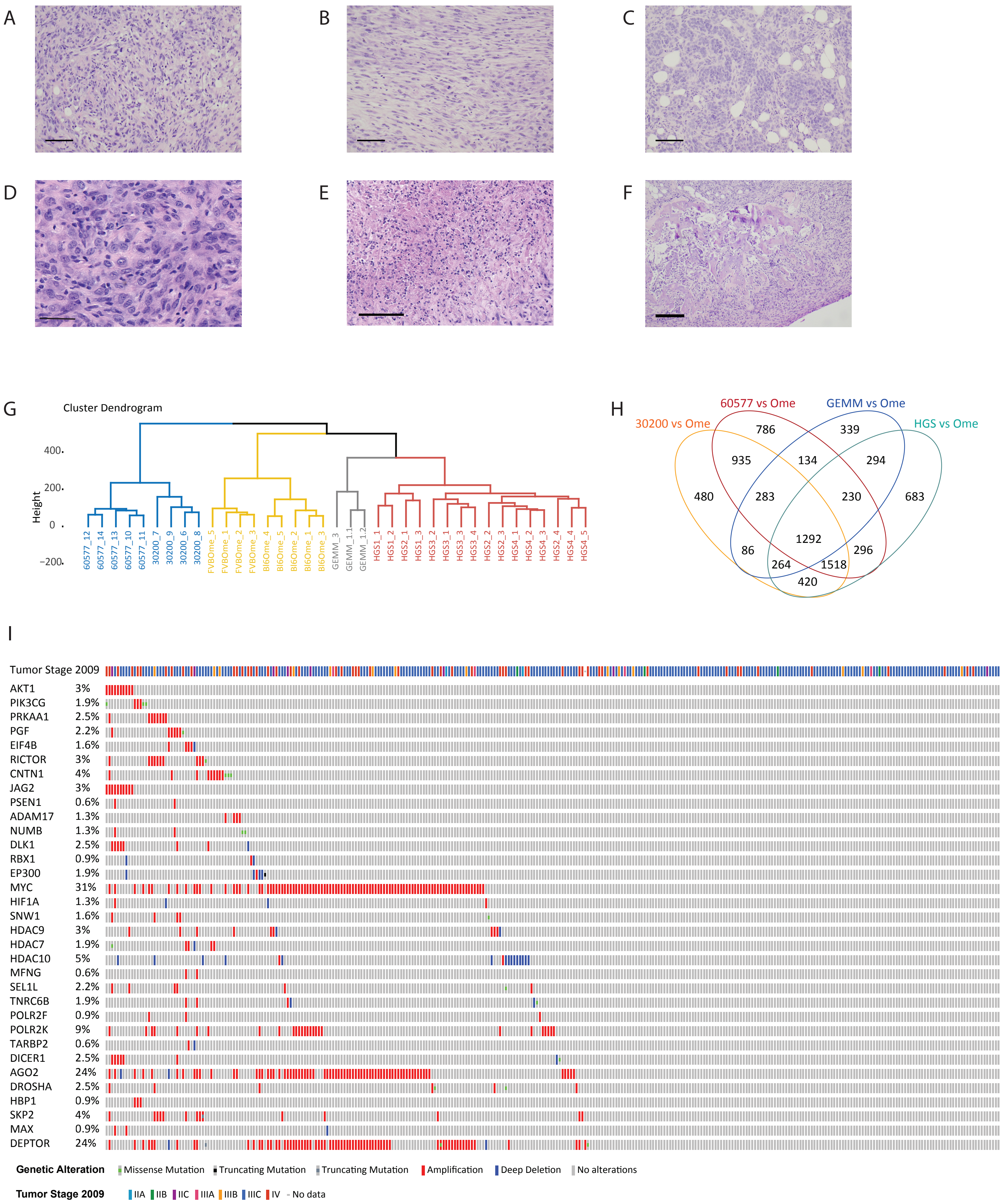

A
B
C
D
E
F
G
Cluster Dendrogram
400
200
0
-200
Height
60577_12
60577_14
60577_13
60577_10
60577_11
30200_7
30200_9
30200_6
30200_8
FVBOme_5
FVBOme_1
FVBOme_2
FVBOme_3
Bl6Ome_4
Bl6Ome_5
Bl6Ome_2
Bl6Ome_1
Bl6Ome_3
GEMM_3
GEMM_1.1
GEMM_1.2
HGS1_1
HGS1_2
HGS2_1
HGS1_3
HGS3_2
HGS3_1
HGS3_3
HGS3_4
HGS2_2
HGS2_3
HGS4_1
HGS4_2
HGS4_3
HGS2_4
HGS4_4
HGS4_5
H
60577 vs Ome
GEMM vs Ome
30200 vs Ome
HGS vs Ome
786
339
935
134
294
480
283
230
683
1292
86
296
264
1518
420
I
Tumor Stage 2009
AKT1 3%
PIK3CG 1.9%
PRKAA1 2.5%
PGF 2.2%
EIF4B 1.6%
RICTOR 3%
CNTN1 4%
JAG2 3%
PSEN1 0.6%
ADAM17 1.3%
NUMB 1.3%
DLK1 2.5%
RBX1 0.9%
EP300 1.9%
MYC 31%
HIF1A 1.3%
SNW1 1.6%
HDAC9 3%
HDAC7 1.9%
HDAC10 5%
MFNG 0.6%
SEL1L 2.2%
TNRC6B 1.9%
POLR2F 0.9%
POLR2K 9%
TARBP2 0.6%
DICER1 2.5%
AGO2 24%
DROSHA 2.5%
HBP1 0.9%
SKP2 4%
MAX 0.9%
DEPTOR 24%
Genetic Alteration
Missense Mutation
Truncating Mutation
Truncating Mutation
Amplification
Deep Deletion
No alterations
Tumor Stage 2009
IIA
IIB
IIC
IIIA
IIIB
IIIC
IV
No data

Figure S1. Histological and molecular characterisation of murine HGSOC models, related to Figure 1. **A-F** Tissue sections were derived from mouse model tumors and stained with hematoxylin and eosin **A**: HGS2, representation of tumour cells organised in sheets, scale bar set to 200µm; **B**: HGS3, representation of spindle-like morphology, scale bar set to 200µm; **C**: 60577, representation of tumour cells organized in nests, scale bar set to 200µm; **D**: HGS2, representation of pleomorphic cells with nuclei with prominent nucleoli, scale bar set to 50µm; **E**: HGS2, necrotic area, scale bar set to 200µm; **F**: HGS2, calcification, scale bar set to 200µm. **G**, Hierarchical cluster analysis of murine sample transcriptomes. **H**, Overlap of differentially-expressed genes (FDR < 0.05) in murine tumours vs normal omental samples. **I,** OncoPrint showing list of genes with high mutation frequency in TCGA and present in CNA regions in mouse models. Annotation of tumour stage has been included.

Figure S2

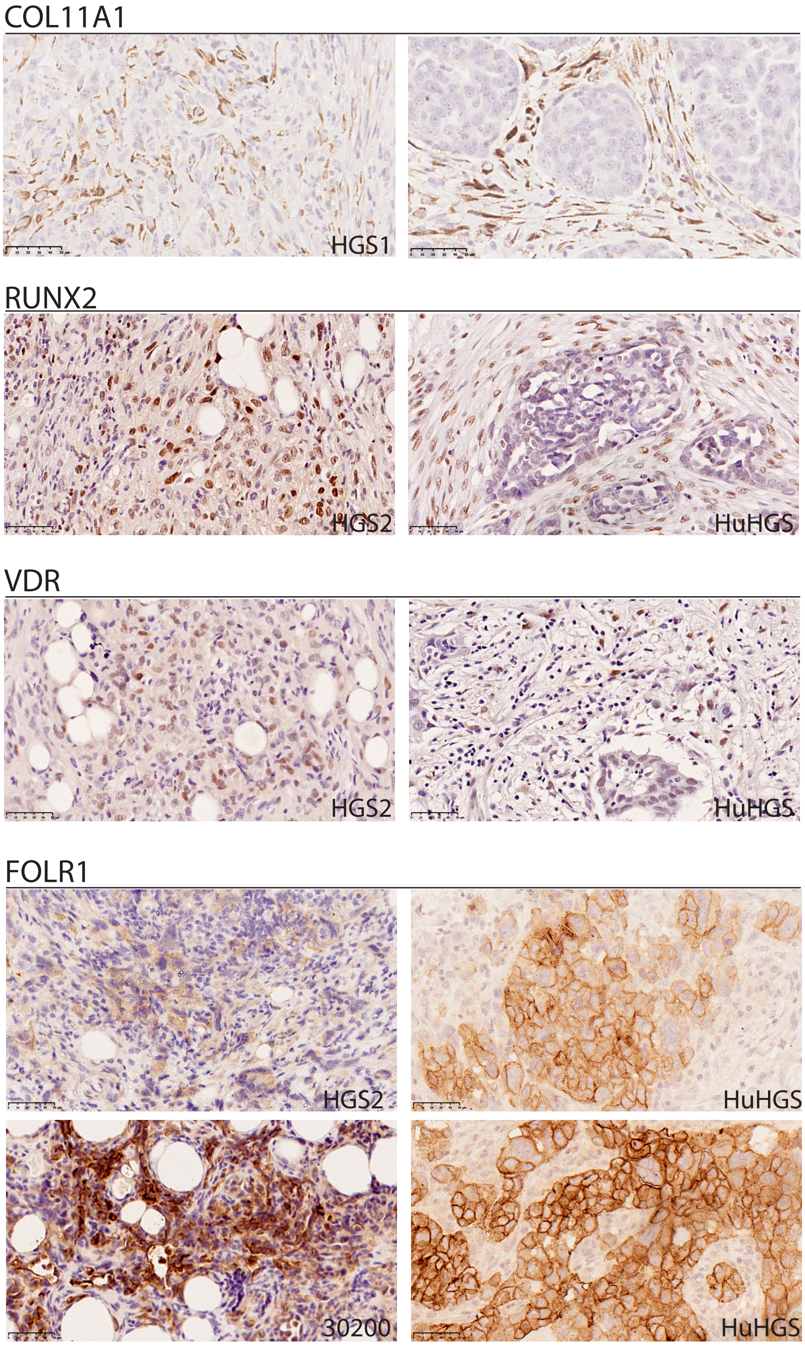
COL11A1
HGS1
RUNX2
HGS2
HuHGS
VDR
HGS2
HuHGS
FOLR1
HGS2
HuHGS
30200
HuHGS

Figure S2. Concordance of murine and human HGSOC tumor-associated proteins, related to Figure 2. **A,** IHC for COL11A1, RUNX2, VDR and FOLR1 staining. Representative images for HGS2 and HuHGS are depicted. For FOLR1, staining on the 30200 model, in which we observed the highest expression of the marker, has been included. Scale bars,100µm (COL11A1) and 50µm, (RUNX2, VDR, FOLR1).

Figure S3

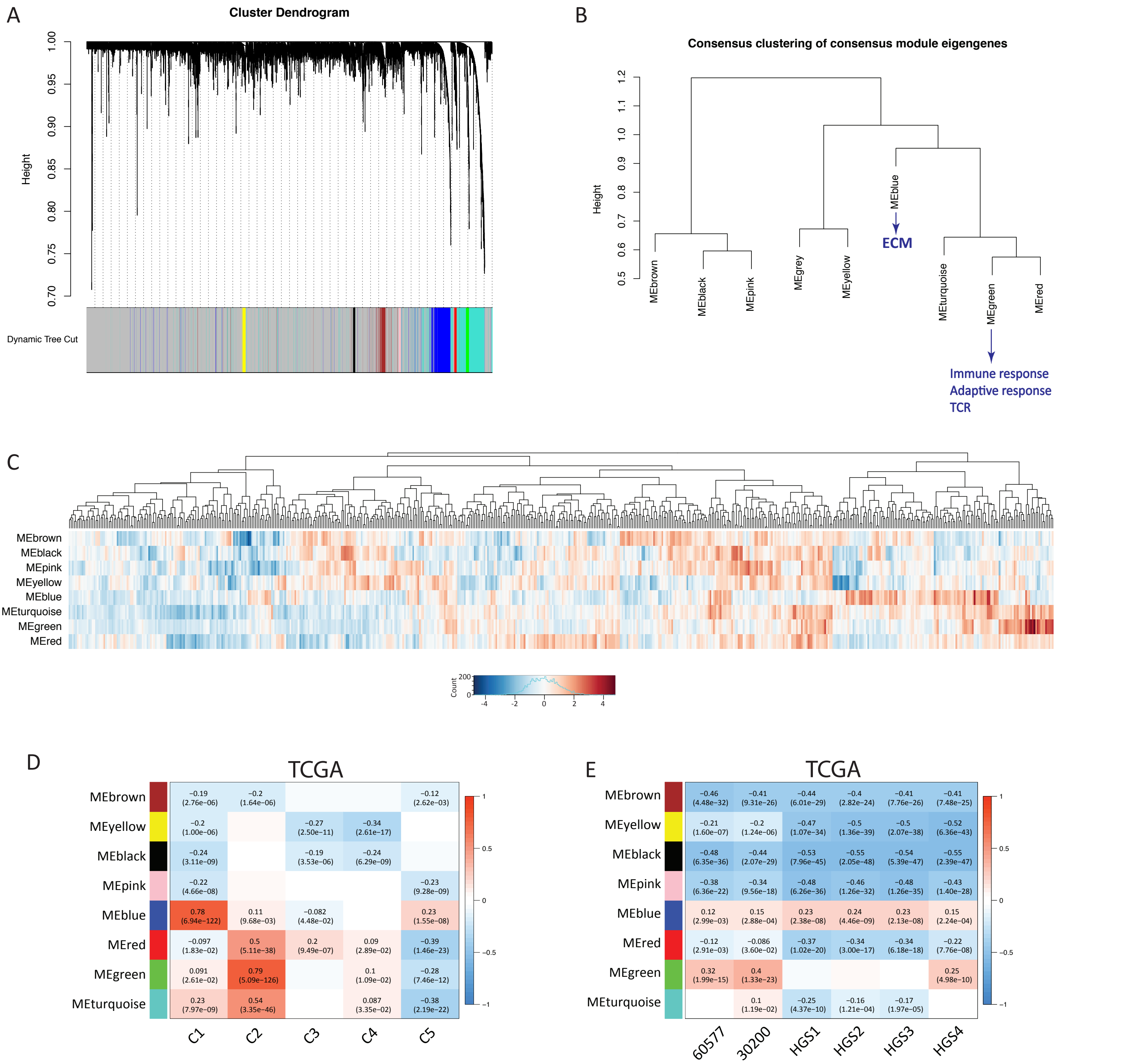
A
Cluster Dendrogram
Height
1.00
0.95
0.90
0.85
0.80
0.75
0.70
Dynamic Tree Cut
B
Consensus clustering of consensus module eigengenes
Height
1.2
1.1
1.0
0.9
0.8
0.7
0.6
0.5
MEbrown
MEblack
MEpink
MEgrey
MEyellow
MEblue
ECM
MEturquoise
MEgreen
MEred
Immune response
Adaptive response
TCR
C
MEbrown
MEblack
MEpink
MEyellow
MEblue
MEturquoise
MEgreen
MEred
Count
200
0
-4
-2
0
2
4
D
TCGA
MEbrown: −0.19 (2.76e−06), −0.2 (1.64e−06), −0.12 (2.62e−03)
MEyellow: −0.2 (1.00e−06), −0.27 (2.50e−11), −0.34 (2.61e−17)
MEblack: −0.24 (3.11e−09), −0.19 (3.53e−06), −0.24 (6.29e−09)
MEpink: −0.22 (4.66e−08), −0.23 (9.28e−09)
MEblue: 0.78 (6.94e−122), 0.11 (9.68e−03), −0.082 (4.48e−02), 0.23 (1.55e−08)
MEred: −0.097 (1.83e−02), 0.5 (5.11e−38), 0.2 (9.49e−07), 0.09 (2.89e−02), −0.39 (1.46e−23)
MEgreen: 0.091 (2.61e−02), 0.79 (5.09e−126), 0.1 (1.09e−02), −0.28 (7.46e−12)
MEturquoise: 0.23 (7.97e−09), 0.54 (3.35e−46), 0.087 (3.35e−02), −0.38 (2.19e−22)
C1
C2
C3
C4
C5
1
0.5
0
−0.5
−1
E
TCGA
MEbrown: −0.46 (4.48e−32), −0.41 (9.31e−26), −0.44 (6.01e−29), −0.4 (2.82e−24), −0.41 (7.76e−26), −0.41 (7.48e−25)
MEyellow: −0.21 (1.60e−07), −0.2 (1.24e−06), −0.47 (1.07e−34), −0.5 (1.36e−39), −0.5 (2.07e−38), −0.52 (6.36e−43)
MEblack: −0.48 (6.35e−36), −0.44 (2.07e−29), −0.53 (7.96e−45), −0.55 (2.05e−48), −0.54 (5.39e−47), −0.55 (2.39e−47)
MEpink: −0.38 (6.36e−22), −0.34 (9.56e−18), −0.48 (6.26e−36), −0.46 (1.26e−32), −0.48 (1.26e−35), −0.43 (1.40e−28)
MEblue: 0.12 (2.99e−03), 0.15 (2.88e−04), 0.23 (2.38e−08), 0.24 (4.46e−09), 0.23 (2.13e−08), 0.15 (2.24e−04)
MEred: −0.12 (2.91e−03), −0.086 (3.60e−02), −0.37 (1.02e−20), −0.34 (3.00e−17), −0.34 (6.18e−18), −0.22 (7.76e−08)
MEgreen: 0.32 (1.99e−15), 0.4 (1.33e−23), 0.25 (4.98e−10)
MEturquoise: 0.1 (1.19e−02), −0.25 (4.37e−10), −0.16 (1.21e−04), −0.17 (1.97e−05)
60577
30200
HGS1
HGS2
HGS3
HGS4
1
0.5
0
−0.5
−1

Figure S3. ICGC and TCGA consensus WGCNA, related to Figure 3. **A,** WGCNA consensus analysis was performed using the ovarian ICGC and TCGA expression datasets and consensus clusters of co-regulated genes are presented as a dendrogram. Colors show different modules (gene programs). **B**, Cluster dendrogram of consensus module eigenvalues (ME) illustrates clustering of programs associated with ECM (MEblue) and immune response (MEgreen) pathways. **C**, Heatmap of consensus ME across TCGA samples (N = 593). **D**, Heatmap of association of C1–C5 classification. Positive associations are shown in red and negative are shown in blue. Pearson's r and p-values are indicated in the fields where a significant association was observed ($p < 0.05$). **E**, Heatmap of association of differentially-expressed gene scores in mouse models. Positive associations are shown in red and negative are shown in blue. Pearson's r and p-values are indicated in the fields where a significant association was observed ($p < 0.05$).

Figure S4

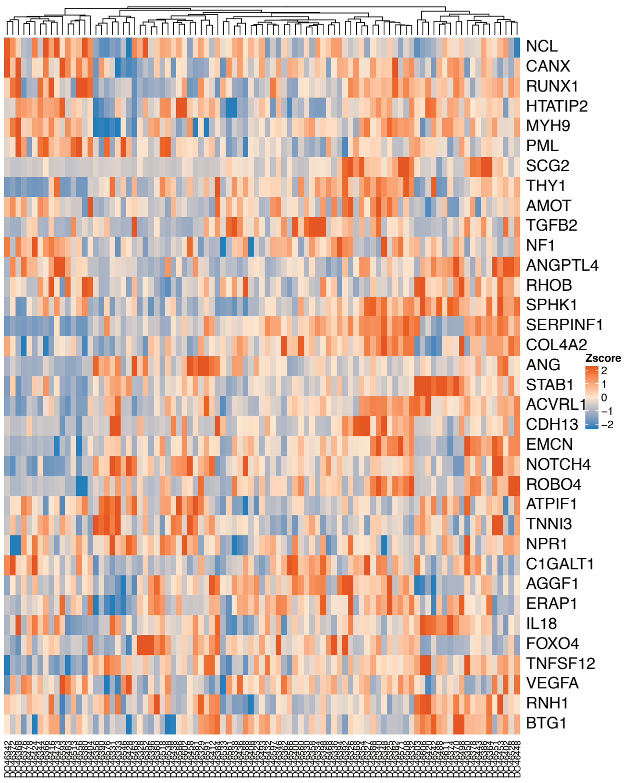
NCL
CANX
RUNX1
HTATIP2
MYH9
PML
SCG2
THY1
AMOT
TGFB2
NF1
ANGPTL4
RHOB
SPHK1
SERPINF1
COL4A2
ANG
STAB1
ACVRL1
CDH13
EMCN
NOTCH4
ROBO4
ATPIF1
TNNI3
NPR1
C1GALT1
AGGF1
ERAP1
IL18
FOXO4
TNFSF12
VEGFA
RNH1
BTG1
Zscore
2
1
0
−1
−2

Figure S4, related to Figure 4. Heatmap illustrating expression of angiogenesis-associated genes (N = 93) across primary HGSOC tumors in the ICGC dataset.

Figure S5

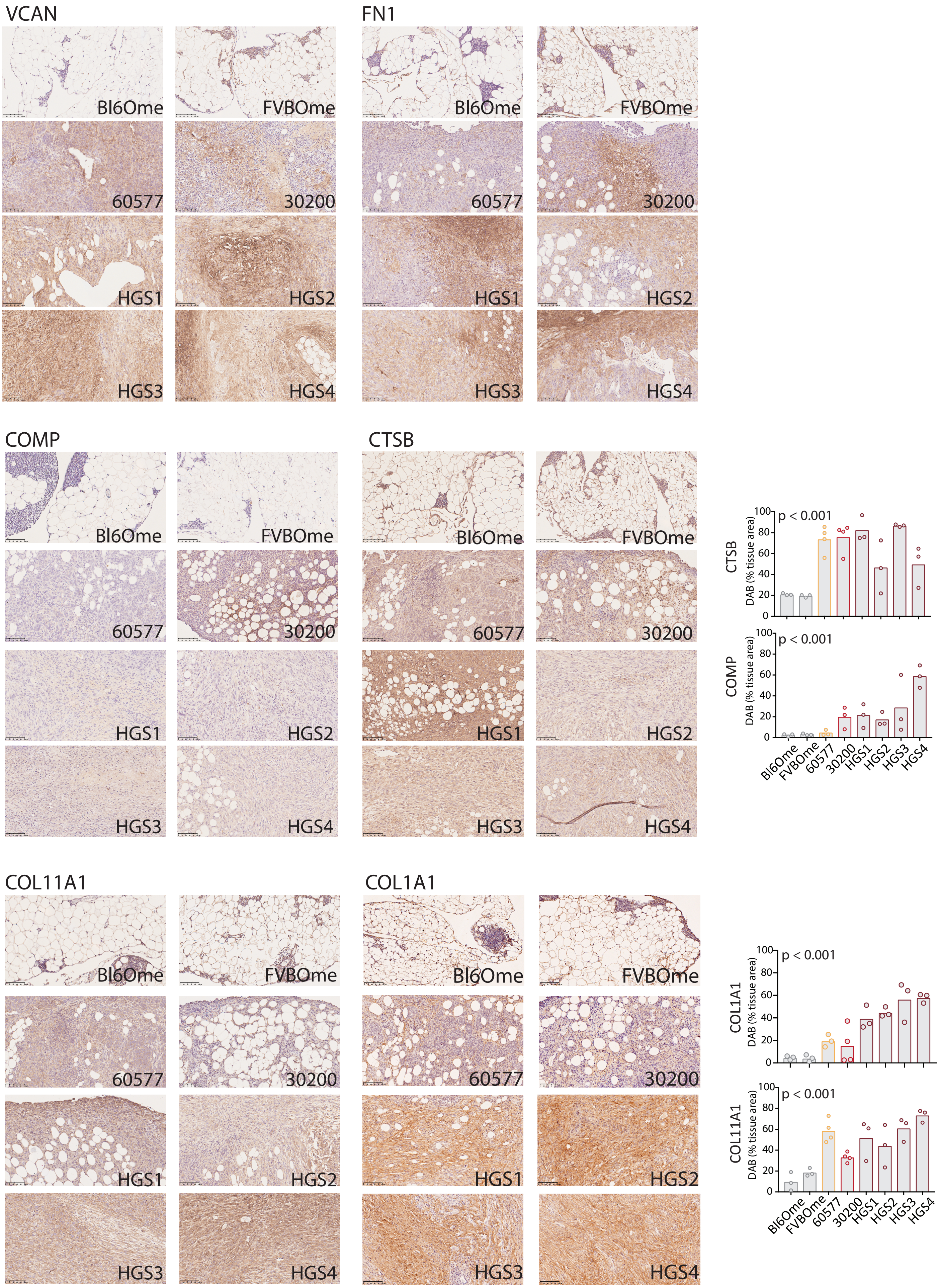
VCAN
FN1
COMP
CTSB
COL11A1
COL1A1
Bl6Ome
FVBOme
60577
30200
HGS1
HGS2
HGS3
HGS4
p < 0.001
DAB (% tissue area)
CTSB
COMP
COL1A1
COL11A1
0
20
40
60
80
100

Figure S5. Matrix index proteins in HGSOC murine models, related to Figure 5. IHC for VCAN, FN1, COMP, CTSB, COL11A1, COL1A1. Representative images all six HGSOC murine models and normal omental tissues is shown. Scale bars,100µm. Barplots illustrate the result of digital analysis and quantification of % positive area by Definiens Tissue Studio®. P-values correspond to one-way ANOVA. Each dot represents a tumor from an individual mouse.

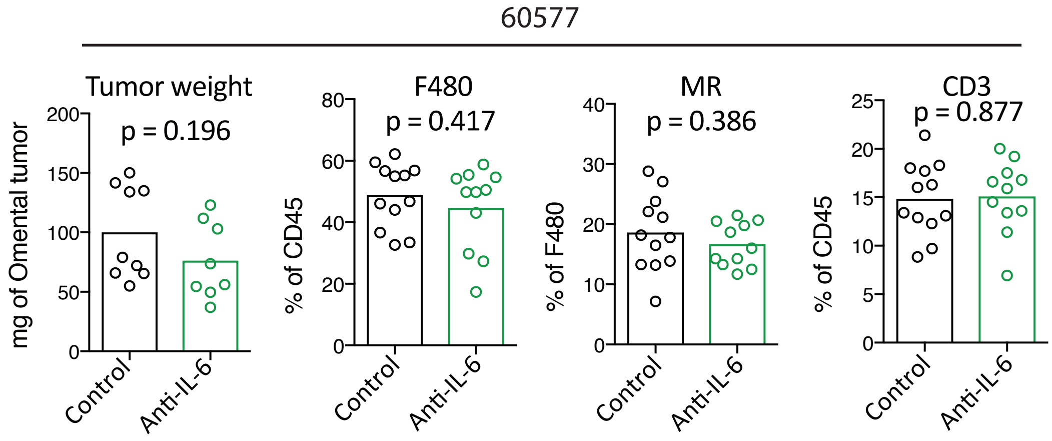
60577
Tumor weight
p = 0.196
mg of Omental tumor
200
150
100
50
0
Control
Anti-IL-6
F480
p = 0.417
% of CD45
80
60
40
20
0
Control
Anti-IL-6
MR
p = 0.386
% of F480
40
30
20
10
0
Control
Anti-IL-6
CD3
p = 0.877
% of CD45
25
20
15
10
5
0
Control
Anti-IL-6

Figure S6. Anti-IL6 treatment of the 60577 murine model, related to Figure 6. Mice injected with 60577 cells were treated with isotype control or anti-IL-6 2 mg/kg i.p. twice weekly, starting 10 days after cell injection until endpoint. The immune infiltrates were analyzed by flow cytometry. Student's t-test values are depicted on the barplots. Each dot represents a tumor from an individual mouse.
